# Microbiome properties in the root nodules of *Prosopis cineraria,* a leguminous desert tree

**DOI:** 10.1128/spectrum.03617-23

**Published:** 2024-04-16

**Authors:** Rashid Ali, Srinivasa R. Chaluvadi, Xuewen Wang, Khaled M. Hazzouri, Naganeeswaran Sudalaimuthuasari, Mohammed Rafi, Mariam Al-Nuaimi, Shina Sasi, Eric Antepenko, Jeffrey L. Bennetzen, Khaled M. A. Amiri

**Affiliations:** 1 Mitrix Bio., Inc., Farmington, Connecticut, USA; 2 Department of Genetics, University of Georgia, Athens, Georgia, USA; 3 Khalifa Center for Genetic Engineering and Biotechnology, United Arab Emirates University, Al-Ain, UAE; 4 Department of Biology, College of Science, United Arab Emirates University, Al-Ain, UAE; Beijing Forestry University, Beijing, China

**Keywords:** Caesalpinioideae, metagenome, metatranscriptome, nitrogen fixation, root nodulation

## Abstract

We conducted a comprehensive analysis of the total microbiome and transcriptionally active microbiome communities in the roots and root nodules of *Prosopis cineraria*, an important leguminous tree in arid regions of many Asian countries. Mature *P. cineraria* trees growing in the desert did not exhibit any detected root nodules. However, we observed root nodules on the roots of *P. cineraria* growing on a desert farm and on young plants growing in a growth chamber, when inoculated with rhizosphere soil, including with rhizosphere soil from near desert tree roots that had no nodules. Compared to nearby soil, non-nodulated roots were enriched with Actinobacteria (e.g., *Actinophytocola* sp.), whereas root nodules sampled from the desert farm and growth chamber had abundant Alphaproteobacteria (e.g., *Ensifer* sp.). These nodules yielded many microbes in addition to such nitrogen-fixing bacteria as *Ensifer* and *Sinorhizobium* species. Significant differences exist in the composition and abundance of microbial isolates between the nodule surface and the nodule endosphere. Shotgun metagenome analysis of nodule endospheres revealed that the root nodules comprised over 90% bacterial DNA, whereas metatranscriptome analysis showed that the plant produces vastly more transcripts than the microbes in these nodules. Control inoculations demonstrated that four out of six *Rhizobium, Agrobacterium,* or *Ensifer* isolates purified from *P. cineraria* nodules produced nodules in the roots of *P. cineraria* seedlings under greenhouse conditions. The best nodulation was achieved when seedlings were inoculated with a mixture of those bacterial strains. Though root nodulation could be achieved under water stress conditions, nodule number and nodule biomass increased with copious water availability. .

**IMPORTANCE:**

Microbial communities were investigated in roots and root nodules of *Prosopis cineraria*, a leguminous tree species in arid Asian regions that is responsible for exceptionally important contributions to soil fertility in these dramatically dry locations. Soil removed from regions near nodule-free roots on these mature plants contained an abundance of bacteria with the genetic ability to generate nodules and fix nitrogen but did not normally nodulate in their native rhizosphere environment, suggesting a very different co-evolved relationship than that observed for herbaceous legumes. The relative over-expression of the low-gene-density plant DNA compared to the bacterial DNA in the nodules was also unexpected, indicating a very powerful induction of host genetic contributions within the nodule. Finally, the water dependence of nodulation in inoculated seedlings suggested a possible link between early seedling growth (before a deep root system can be developed) and the early development of nitrogen-fixing capability.

## INTRODUCTION

Plants of the Arabian Desert are subject to a host of environmental challenges, including extremes of heat, aridity, and very low soil fertility. A native tree species, *Prosopis cineraria* (Family: Fabaceae, Subfamily: Caesalpinioideae, Clade: Mimosoid, Common name: Ghaf), thrives and reproduces in this extreme environment. It is also the dominant woody tree species in the arid deserts of many countries in West Asia, the Middle East, and the Indian Subcontinent (1, 2). *P. cineraria* is a phreatophyte that can produce a very long (>10 m) taproot to allow it to utilize water from underground aquifers (1, 3, 4).

The *Prosopis* species produce indeterminate root nodules containing potential nitrogen-fixing rhizobial species (5). *P. glandulosa* was found to be nodulated by a diverse collection of microbes such as *Rhizobium meliloti*, *Rhizobium leguminosarum* bv. phaseoli, *Parasponia,* and *Bradyrhizobium* sp. (3). In greenhouse experiments, *Ensifer* and *Mesorhizobium* isolates nodulated *P. farcta*, and *Ensifer meliloti* was the most effective, as measured by nodule number and plant biomass (6). Three *E. fredii* strains isolated from *Vachellia gummifera* could nodulate *Prosopis chilensis* and other plant species, such as *Acacia cyanophylla* and *Leucaena leucocephala* (7).

The root nodules of leguminous plants are inhabited by both symbiotic nitrogen-fixing rhizobia (e.g., members of the genera *Rhizobium*, *Bradyrhizobium*, *Sinorhizobium*/*Ensifer*) and non-rhizobial Proteobacteria (e.g., *Bacillus*) and Actinobacteria (8). Some of these non-rhizobia may play a role in nodulation or post-nodulation functions. For instance, co-inoculation of rhizobia and *Bacillus* strains was found to increase chickpea yield by 22% compared to inoculation with rhizobia alone in field experiments (9). *Paenibacillus* isolates from *Medicago sativa* nodules exhibit chitinolytic activity, which is useful in removing rhizobial lipochitooligosaccharide (nod factor) signaling molecules produced by the bacteria for plant:rhizobia communication, but are not required for the progression of an infection thread (10). Some bacteria, such as strains of *Agrobacterium rhizogenes,* improved nodulation in clover when co-inoculated with a rhizobial strain (11). Other non-nitrogen-fixing bacteria in the nodule may be commensals, which are common in any complex microbial system (8, 12).

Rhizobia contain a multipartite genome consisting of a single chromosome and one or two mega plasmids. Many symbiotic and nitrogen fixation genes are routinely found on the symbiotic plasmids and occasionally in the core genome, also called symbiotic islands (13, 14). The non-symbiotic plasmids, whenever present, have genes that indirectly benefit symbiosis, such as bacteroid development, carbon uptake, a free-living lifestyle, and abiotic stress tolerance (15, 16). Nodule formation and symbiosis require the coordinated function of more than 200 plant genes (17) and about 73 bacterial genes (18).

We have recently completed the full-genome sequence of *P. cineraria* (19), thus permitting a complete description of the activities of all the genes in this plant. This manuscript analyzes nodule microbiome communities and their metagenomes and metatranscriptomes. We also described the isolation of bacteria from the nodules and surrounding soil. These isolated microbes were examined for their ability to produce nodules on *P. cineraria* roots. Our results discover and describe the great complexity in composition, roles, and overall activities of the many participants in this plant:microbe interaction.

## RESULTS

In the current study, we examined the composition of total and active microbiomes in the roots and root nodules of *P. cineraria* growing in three environments, including (i) an open desert without supplemental irrigation, (ii) a desert farm under irrigation, and (iii) a growth chamber. We analyzed microbiomes by various methods, including (i) 16 rRNA gene sequence diversity, (ii) *nif*H gene sequence diversity, (iii) shotgun metagenomic analysis, and (iv) shotgun metatranscriptome analysis. Additionally, we purified and evaluated some bacteria present in the root nodules for their ability to produce root nodules and promote growth of *P. cineraria* seedlings under greenhouse conditions.

### Nodulation in the roots of *P. cineraria* trees was associated with a taxonomic shift in the root-associated microbiome

We analyzed microbial diversity in the roots and nodules of two *P. cineraria* populations in the Al Ain Desert in UAE. We sampled the soil and roots of three mature trees growing in the open desert and the roots and nodules from three nodulating trees growing in a polyhouse tunnel in a desert farm where the trees are frequently irrigated. The three desert trees, and all other mature desert trees that we investigated, did not exhibit any root nodules that we detected (Fig. S1A), whereas those growing in the desert farm had abundant root nodules (Fig. S1B and C).

To focus on important, our first analysis was of 16S amplicons. The 16S amplicon sequence diversity, as expressed by the Shannon-diversity index, was higher in the root ectosphere and endosphere (REE) compared to rhizosphere soil (Rh) and bulk soil (Fig. S2A). Rh fraction has the lowest microbial diversity (Fig. S2A). Similarly, in the nodules, the nodule surface microbiome (NSM) has higher microbial diversity than the nodule endosphere microbiome (NEM) (Fig. S2B). The NSM fraction has the highest microbial diversity compared to NEM, REE, Rh, and bulk soil.

**Fig 1:**
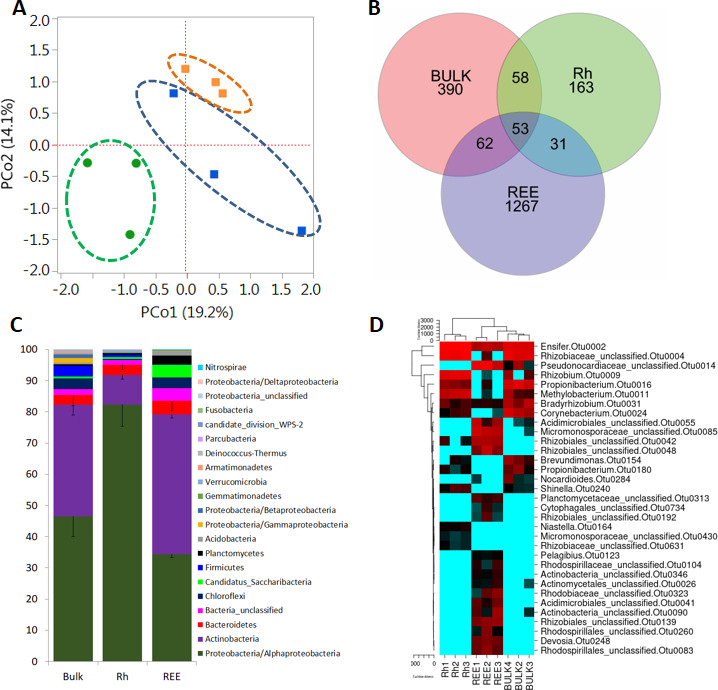
Bacterial diversity in the rhizospheres and roots of *P. cineraria* trees growing in the open desert. (**A**) PCoA plots of bacterial OTUs from the roots and rhizospheres, generated from a distance matrix for the Jaccard indices set using a rarified 16S OTU table. Plots show the first two principal axes. Rhizosphere soil samples are depicted by orange squares, bulk soil (BULK) samples are shown in blue squares, and root samples (REE) are represented by green circles. (**B**) Venn diagrams showing shared and unique OTUs from bulk soil, Rh, and REE. (**C**) The relative abundances of bacterial phyla in the bulk soil, Rh, and REE. The averages of each phylum for all samples of bulk soil, Rh, and REE were shown as three column bars. Error bars are derived from three independent biological replicates. (**D**) A hierarchical cluster analysis of relative OTU abundance (Y-axis) of significant OTUs versus sample groups bulk soil, Rh, and REE used in the current study. Red indicates high abundance and blue indicates low abundance.

Principal coordinate analyses (PCoA) of Bray-Curtis distances revealed distinct clustering of desert tree samples from roots, rhizosphere, and soils (Fig. 1A). REE samples have more unique operational taxonomic units (OTUs) compared to Rh and bulk soil fractions (Fig. 1B), with very few (a mere 53) shared by all three compartments but including *Ensifer* OTUs (Fig. 1B and D). The abundances of different phyla and genera were assessed by taxonomic assignment using the RDP classifier and comparative analysis using MOTHUR tools. At the phylum level, Alphaproteobacteria and Actinobacteria were most abundant phyla in all sample types. Actinobacteria and a relatively unknown bacterium, *Candidatus_Saccharibacteria,* were abundant and significantly enriched in the REE fraction (Fig. 1C). Out of 3,899 OTUs, 35 OTUs showed significantly different enrichment between Bulk, Rh, and REE samples. A heatmap analysis of these significant OTUs showed two distinct clusters, with Rh samples forming one cluster and BULK and REE samples forming the other. Bulk soil samples were included in a subcluster within the second cluster (Fig. 1D). For example, some of the dominant OTUs such as OTU2 (*Ensifer* sp.), OTU4 (Rhizobiaceae_unclassified), OTU9 (*Rhizobium* sp.), OTU11 (*Methylobacterium* sp.), and OTU16 (*Propionibacterium* sp.) are significantly underrepresented in the REE fraction. Many actinobacterial members, such as OTU14, OTU26, OTU41, OTU55, OTU90, and OTU346, are overrepresented in the REE fraction (Table S1). The phylogeny of some actinobacterial OTUs which are related to known actinobacteria is shown in Fig. S6.

*P. cineraria* trees growing on a desert farm under irrigation produced abundant root nodules. A PCoA plot depicting the differences in nodule microbiome composition showed loose clustering of samples from nodule surface and nodule endosphere (Fig. S3A). The NSM fraction had a richer diversity, containing 2,824 unique OTUs, in contrast to the NEM fraction, which had only 317 unique OTUs (as depicted in Fig. S3B). Among the 18 microbial phyla identified within the nodules, Alphaproteobacteria, Actinobacteria, Bacteroidetes, and Planctomycetes stood out as the most prevalent, together accounting for 90% of the sequence data. Alphaproteobacteria was more abundant in the NEM fraction, whereas Bacteroidetes are more abundant in the NSM fraction (Fig. S3C). Out of 3,665 OTUs found in the nodules, 45 OTUs showed a significant difference in their relative abundances between the two compartments. The heatmap analysis of significant OTUs separated these OTUs into two distinct clusters, representing either NSM or NEM. Many bacterial OTUs (blue) are significantly absent or occur in very low abundance in the nodule endosphere fraction (Fig. S3D). Among the most abundant OTUs, OTU2 (*Ensifer* sp.), OTU4 (Rhizobiaceae_unclassified), OTU7 (*Rhizobium* sp.), OTU9 (*Rhizobium* sp.), and OTU12 (*Chitinophaga* sp.) showed significant enrichment in the nodules but not in the roots without nodules. However, OTU14 (*Actinophytocola* sp.) was more abundant in the roots than in the nodules (Table S2).

### Nodule microbiome from *P. cineraria* seedlings grown in a growth chamber showed nodule compartment-specific and genotype-specific enrichment

To gain a deeper understanding of the microbiome composition under controlled conditions, we grew *P. cineraria* plants using seeds collected from trees growing in the open desert (DT) and desert farm (FT). The seeds were germinated in a 1:1 mix of potting soil and the rhizosphere soil collected from the *P. cineraria* tree growing in the desert. After 5 weeks, we collected multiple root nodules of various sizes from four FT plants and three DT plants (Fig. S4) and isolated metagenomic DNA from nodule surface and nodule endosphere for each nodule. Ultra-thin section of the fresh nodule indicated the presence of bacteroids in many, but not all, cortex cells (Fig. S5A). Individual nodule cells exhibited membrane-bound bacteroids under a transmission electron microscope (Fig. S5B). Metagenomic analyses were then performed to determine total microbiome composition and candidate nitrogen-fixing microbiome assessed by 16S gene and *nif*H gene sequence diversity. An OTU file obtained from 71 individual nodule samples was used for the final 16S bacterial and *nif*H analyses. The current investigation resulted in a total of 734 16S OTUs and 72 *nif*H OTUs.

#### 16S microbiome diversity

**Fig 2:**
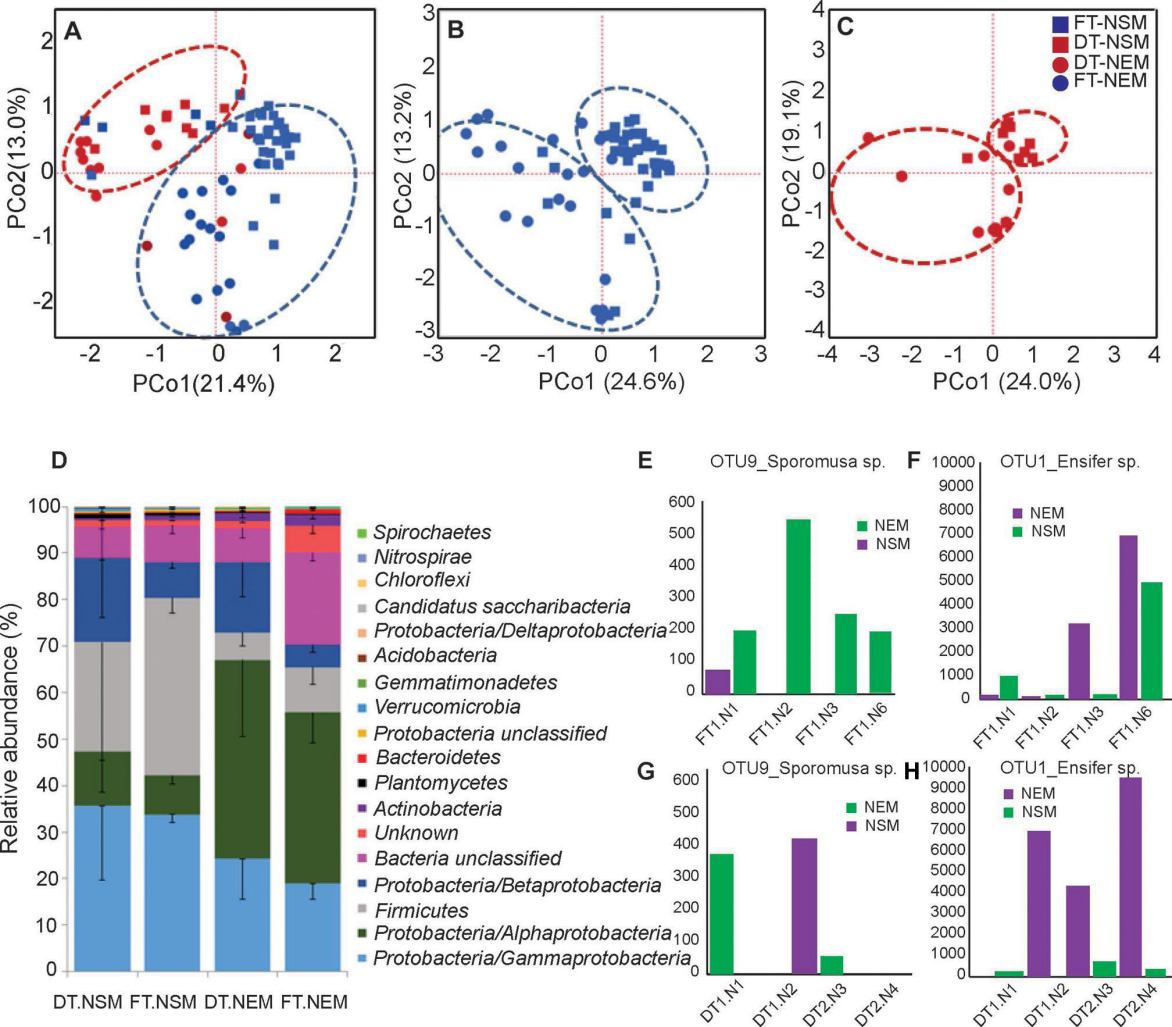
Microbial diversity in the root nodules of growth chambers grown seedlings of *P. cineraria*. (A) Principal coordinates analysis of bacterial OTUs from the root nodules of *Prosopis cineraria* growing in the growth chambers. PCoA plots were generated from a distance matrix for the Jaccard indices set using the rarified 16S OTU table. Plots show the first two principal axes. Nodules from DT tree seedlings are shown in red, and FT tree seedlings are shown in blue. Nodule Surface Microbiome (NSM) samples are depicted with squares, and Nodule Endophytic Microbiome (NEM) samples are shown with circles. (B) PCoA plot showing NSM and NEM samples from FT tree seedlings. (C) PCoA plot showing NSM and NEM samples from DT tree seedlings. (D) Relative abundance of bacterial phyla in the NSM and NEM compartments of root nodules collected from DT seedlings and FT seedlings. The error bars were derived from at least 10 biological replicates. The relative abundance of OTU9 (*Sporomusa* sp.) and OTU1 (*Ensifer* sp.) in nodules of a single FT plant (E, F) and a single DT plant (G, H). The smallest to largest nodules are labeled N1 to N6.

The PCoA graph in Fig. 2A compares bacterial communities in the NSM and NEM compartments of two different *P. cineraria* genotypes (tree seed sources) inoculated with the same soil sample. We observed significant genotype-dependent separation of microbiome communities. When samples from each genotype were separately analyzed, the samples within the genotypes showed nodule compartment-specific separation (Fig. 2B and C). A non-parametric analysis (AMOVA) showed statistically significant differences in bacterial diversity between genotypes (*P*-value ~0.001). A total of 127 genera from 15 phyla were represented by more than 100 reads in the Illumina data set. The phyla of Proteobacteria (53.3%), Firmicutes (15.4%), Actinobacteria (2.6%), and Unclassified bacteria (23.3%) contributed 94.6% of the total bacterial diversity. The subphylum Alphaproteobacteria were the most abundant in the endosphere compartment. In contrast, Gammaproteobacteria were the most abundant class on the nodule surface (Fig. 2D). Firmicutes are also more abundant on the nodule surface than in the nodule endosphere (Fig. 2D). Figure 2E through H shows some examples of host-genotype-dependent differences in bacterial abundances. Figure 2E and G depict OTU9 (Phylum: Firmicutes, Genus: *Sporomusa* sp.) with greatest abundance on the nodule surface in the FT genotype. In contrast, *Ensifer* OTU1 is the most dominant in the NEM compartment but much more so in the DT genotype, and its abundance increased with the size of the nodule (Fig. 2F and H, and data not shown).

### Shotgun metagenomic analyses revealed that bacterial DNA constitutes a major portion of all nodule metagenomes

**Fig 3:**
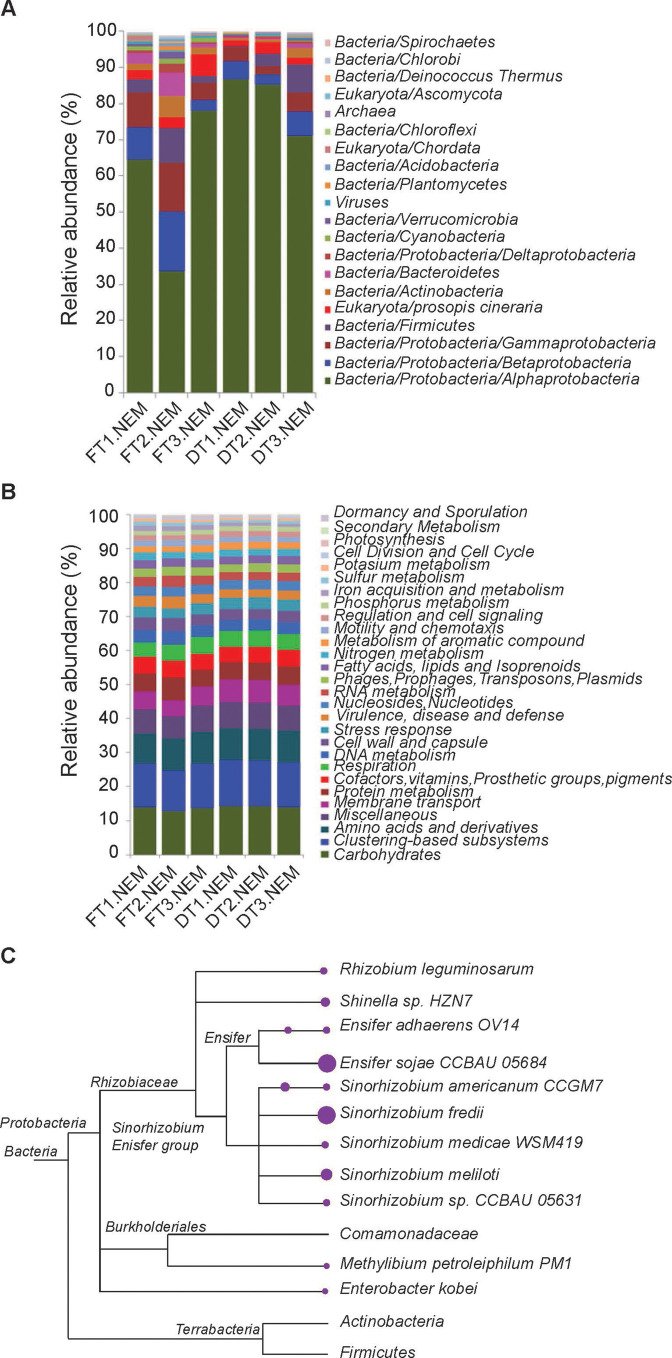
Microbial diversities of nodule microbiomes and their functional potential. (**A**) Relative distribution of the microbial phyla and host DNA in the root nodule metagenomes of *P. cineraria*. The phylum Proteobacteria was further divided into classes (Alpha-, Beta-, Gamma-, and Deltaproteobacteria), and their relative abundance is shown separately. (**B**) Relative distribution of annotated sequence reads on the functional protein subsystem categories in the databases of MG-RAST. (**C**) Phylogenetic presentation of the core microbiomes in the *P. cineraria* nodules, with purple circle size proportional to abundances of the indicated OTUs.

We performed direct Illumina sequencing of the DNA extracted from three DT and three FT samples from single nodules. About 1.75 million to 2.5 million high-quality reads were generated from each sample. The sequence data were quality trimmed and annotated with MG-RAST online server version 4.0.2. All the annotated reads were compared for taxonomic and functional assignments and then used in subsystem reconstructions. Based on the NCBI RefSeq database used in the MG-RAST pipeline, 93.1%–98.1% of these annotated sequences had the closest homology to the genome segments found in bacteria, 1.5%–6.2% to *P. cineraria*, 0.02%–0.5% to fungi, 0.3%–0.6% to viruses, and 0.1%–0.4% to archaea (Fig. 3A). The metagenome sequences were annotated with SEED subsystems-based functions (level 1, level 2, level 3, and function), summed, and then standardized as a function of total reads within each sample. These results are presented in Table S3. We found 28 level 1 subsystems, 166 level 2 subsystems, 1,096 level 3 subsystems, and 7,412 potential metagenome functions. Figure 3B shows the relative abundance of 28 level 1 metagenome subsystems, out of which the carbohydrate subsystem was the most dominant. The top 4 functions enriched in the root nodule metagenomes include adenylate cyclase (EC 4.6.1.1), phage major capsid protein, genes involved in the biosynthesis of lipopolysaccharides, phage capsid and scaffold, and decarboxylase (Table S3). The core microbiome, as determined by MEGAN analysis of NCBI BLAST output, showed that the dominant membership of the core nodule microbiome was Rhizobiaceae (e.g., *Ensifer* sp. and *Sinorhizobium* sp.), and some members belong to Burkholderiaceae, Actinobacteria, and Firmicutes (Fig. 3C).

### Metatranscriptome analyses demonstrated enormous enrichment of host plant transcription relative to the bacteria in root nodules

We utilized ribodepleted RNASeq libraries to examine the transcriptional activity of plant and microbial genomes in the nodules. In Fig. S9A, we compared the relative abundance of total host and microbiome phyla in the DNA and RNA samples taken from DT1, DT2, and DT3 nodules. The results showed that the RNA samples contained a significantly higher proportion of host plant transcripts (82.6%) compared to the DNA samples (6.2%), suggesting that the plant transcriptome physiologically dominates the interaction within the nodules (Fig. S9A), and the genes related to protein metabolism are most abundant in the nodule transcriptome (Fig. S9B). The phylogeny of partially assembled metatranscriptome reads is shown in Fig. S10. Alphaproteobacteria is the most dominant phylum in the nodules. However, the transcription of proteobacteria was, relatively, diminished in the nodules, from 90.2% in the metagenome to 8.7% in the metatranscriptome.

Analysis of ribodepleted libraries indicated that there were far more plant RNA reads than microbial reads, even though microbiome DNA was much higher in the nodules. We found 82.6% of *P. cineraria* transcripts, indicating that the plant genes in the nodule were much more active than the bacterial genes in the nodule. However, an alternative explanation is that our ribodepletion process somehow enriched plant transcripts relative to bacterial transcripts. To investigate this possible artifact, we performed qPCR for several genes from the plant and the bacteria using total RNA (without ribodepletion) as a template. Our qPCR analysis for these seven plant and bacterial genes indicated Ct values (which correlate to the number of target copies in the sample) appear to be in-line with RNAseq results (Table S4).

The top 20 MG-RAST subsystem functions that were significantly more abundant in the nodule metatranscriptomes compared to the metagenomes primarily belonged to protein metabolism, carbohydrates, nitrogen fixation, cluster-based subsystems, respiration, and RNA metabolism categories (Table 1; Table S3).

**TABLE 1:** The microbiome functions that are significantly more abundant in the root nodule endosphere metatranscriptome than in the root nodule metagenomes of *Prosopis cineraria*^*a*^

Function	DT1.NEM.DNA	DT2.NEM.DNA	DT3.NEM.DNA	DT1.NEM.RNA	DT2.NEM.RNA	DT3.NEM.RNA	Significance
GTP-binding protein	0.152	0.168	0.181	1.701	1.471	1.499	*
RNA helicase, putative	0.003	0.007	0.004	1.140	1.145	1.006	*
Nitrogenase (molybdenum-iron) beta chain (EC 1.18.6.1)	0.084	0.100	0.075	0.924	0.799	0.593	*
Cytochrome c oxidase polypeptide I (EC 1.9.3.1)	0.115	0.133	0.128	0.713	0.673	0.780	*
Heat shock protein 60 family chaperone GroEL	0.209	0.217	0.216	0.746	0.761	0.642	*
SSU ribosomal protein S27Ae	0.000	0.000	0.000	0.686	0.547	0.732	*
Nitrogenase (molybdenum-iron) alpha chain (EC 1.18.6.1)	0.077	0.085	0.069	0.726	0.666	0.531	*
Pyruvate kinase (EC 2.7.1.40)	0.065	0.049	0.062	0.639	0.651	0.488	*
Chaperone protein HtpG	0.102	0.108	0.125	0.710	0.438	0.604	*
Decarboxylase	0.545	0.533	0.505	0.593	0.552	0.555	No
Chaperone protein DnaK	0.082	0.080	0.084	0.464	0.543	0.661	*
Kup system potassium uptake protein	0.162	0.163	0.153	0.506	0.558	0.530	*
Thioredoxin	0.112	0.118	0.126	0.534	0.527	0.518	*
Pre-mRNA-splicing factor PRP8	0.000	0.000	0.001	0.523	0.510	0.473	*
Beta-galactosidase (EC 3.2.1.23)	0.144	0.161	0.187	0.488	0.430	0.431	*
Serine hydroxymethyltransferase (EC 2.1.2.1)	0.092	0.102	0.099	0.484	0.423	0.396	*
ATP-energized glutathione S-conjugate pump, putative	0.001	0.002	0.001	0.433	0.419	0.381	*
Phosphoenolpyruvate carboxylase (EC 4.1.1.31)	0.077	0.057	0.070	0.457	0.435	0.333	*
Thiamin biosynthesis protein ThiC	0.096	0.095	0.097	0.469	0.397	0.325	*
Translation elongation factor Tu	0.090	0.094	0.094	0.344	0.335	0.497	*

^*a*^ The significant MG-RAST functions (*) have a *P*-value of <0.001.

Numerous nitrogen fixation-related genes (*nif*) were found to be significantly overexpressed in the metatranscriptomes, belonging to various species such as *Ensifer aridi*, *Sinorhizobium meliloti*, *Sinorhizobium medicae*, *Mesorhizobium loti*, *Burkholderia vietnamiensis*, *Chelativorans* sp. BNC1, *Frankia* sp. CcI3, *Pseudomonas stutzeri, Rhizobium etli*, *Rhodopseudomonas palustris*, *Zymomonas mobilis*, *Dechloromonas aromatica*, *Methylobacterium* sp., *Anabaena variabilis*, and *Nostoc punctiforme*. Additionally, other nodulation-related genes (e.g., *nod* and *fix* genes) identified in the metatranscriptome belonged to *S. meliloti, R. leguminosarum, S. fredii, Bradyrhizobium* sp*., Azorhizobium caulinodans, Agrobacterium tumefaciens, S. medicae*, and *Mesorhizobium loti*.

### Comparison of host plant transcriptomes in the roots and nodules

To understand the gene regulation in the nodule, we further assembled and analyzed transcriptomes in both the nodule and root. The differentially expressed genes (DEGs) were identified as exhibiting at least twofold changes in abundance, and a *P*-value <0.001 with DEseq2 (20). Compared with the transcript abundance in root samples, 1,099 DEGs were identified in nodule samples, out of which 352 genes were significantly upregulated, and 767 genes were downregulated considerably. The functions of the DEGs were annotated against public databases, and Gene Ontology analyses of these DEGs showed that 711 GOs were significantly enriched. Further metabolic pathway analysis revealed that DEGs were mapped into 89 pathways in the KEGG database and 101 pathways in the Biosys database and significantly enriched in 3 KEGG pathways and 9 BioCyc pathways at *P* < 0.01 (Supplementary data, Table S5). The significant KEGG pathways include primary metabolism, plant hormone signal transduction, and biosynthesis of secondary metabolites (Fig. S11). In comparison, the significant BioCyc pathways include caffeoyl glucarate biosynthesis, flavonoid biosynthesis, phenylpropanoid biosynthesis, indole-3-acetate degradation IV, chitin degradation II, indole-3-acetyl-amide conjugate biosynthesis, indole-3-acetate degradation V, and lysine biosynthesis VI. In all, 765 DEGs are downregulated in the root nodules, and 352 DGGs are upregulated (Supplementary data). Many key DEGs involved in plant hormone signal pathways were differentially regulated between the two tissue types (Fig. S12 and S13).

### Controlled inoculation of bacterial isolates from the root nodules of *P. cineraria* showed that only some bacteria could form root nodules and improve plant growth

The purification and characterization of selected nodule-inhabiting bacteria were described in the supplementary information. A total of 23 bacterial isolates were purified, and 16S sequence analysis was employed to identify the genera (and, often, species) of each purified microbe. Of these, 6 isolates turned out to be *Ensifer* sp. and 16 isolates turned out to be *Rhizobium* sp. rhizobia, and their relative phylogenetic relationships are shown in Fig. S14, and their full-length 16S rRNA gene sequences can be found in the supplementary data. Six of these Rhizobiaceae, including one *Agrobacterium* that was purified, were randomly selected to perform seedling root inoculation studies.

**Fig 4:**
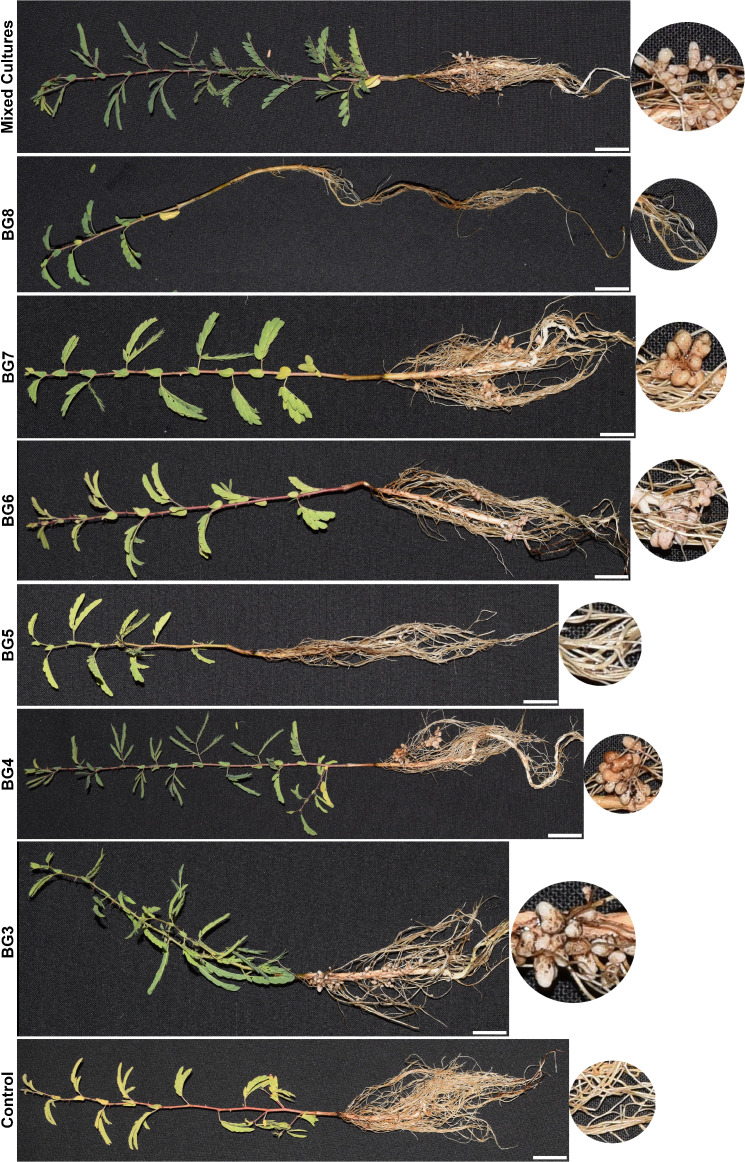
Nodulation efficiency of Rhizobiaceae strains BG3 (*Ensifer meliloti*)*,* BG4 (*Rhizobium* sp. BG4)*,* BG5 (*Agrobacterium fabrum),* BG6 (*Rhizobium* sp. BG6)*,* BG7 (*Rhizobium grahamii), and* BG8 (*Sinorhizobium* sp.). Single inoculations of each individual strain and a mixture of all the six strains were tested.

Individual nodule isolates of nodule-inhabiting proteobacteria were tested for their potential to induce nodulation in *P. cineraria* seedlings (Fig. 4). All strains except BG5 (*Agrobacterium* sp.) and BG8 (*Sinorhizobium* sp.) produced root nodules. Nodule size, shape, and number also varied based on the species of rhizobia tested. The nodule number increased when a mixed culture of six individual isolates was used as inoculum (Fig. 4). A more than 50% increase in nodule number was also observed when the *Agrobacterium* strain (BG5) was co-inoculated with an *Ensifer* strain (BG3) compared to the BG3 strain alone. We also tested the bacterial consortia on other Fabaceae family members, such as cowpea and soybean. No nodulation was observed (data not shown), indicating that the isolated rhizobial species are selective for their host(s).

*P. cineraria* seedlings were inoculated with the above rhizobial mixture, and the control plants without rhizobial inoculation were grown for 7 weeks to test their tissue nitrogen and carbon content. The plants treated with rhizobia were much healthier than the control plants (Fig. S15A). Analysis of carbon and nitrogen in the aboveground biomass of *P. cineraria* plants showed a significant increase in the tissue nitrogen and a significant decrease in the carbon:nitrogen ratio in *P. cineraria* seedlings inoculated with rhizobia. The tissue carbon content was slightly higher than the *P. cineraria* seedlings inoculated with rhizobia, but the difference was not significant (Fig. S15B).

### Water abundance was found to have a major effect on nodule formation in growth chamber studies

*P. cineraria* plants were germinated in pots and grown at 100% field capacity (FC) until the two-leaf stages. Subsequently, the seedlings were inoculated with rhizobial mix and maintained at 14%, 30%, and 100% FC for 7 weeks. The aboveground and belowground portions of the plants were pictured (Fig. S16A and B). The shoot weight, root weight, nodule number, and nodule weight increased with the increase in moisture level from 14% to 30% FC. We observed a significant increase in the shoot weight between plants grown at 30% FC and 100% FC, even though we did not find a significant increase in the root weight, nodule number, and nodule weight between these treatments (Fig. S16C and D).

## DISCUSSION

The Ghaf tree is an important species in its natural environment and is commonly used in desert agroforestry for erosion control, revegetation, and soil improvement. This study focuses on the interaction between the roots of Ghaf trees and microorganisms, specifically examining root nodulation and nitrogen fixation, which are beneficial outcomes unique to Fabaceae. While root nodulation has been extensively studied in annual and perennial legumes (17, 21, 22), there is limited research on the nodule microbiome and factors affecting root nodulation in leguminous trees like *P. cineraria* that grow in arid desert habitats. In contrast to crop plants in agricultural settings, the bacterial diversity on the *P. cineraria* roots (REE) is higher than rhizosphere soil, as previously observed in some other sandy-desert plants (23). Actinobacteria is the dominant phylum in the non-nodulating roots of *P. cineraria* trees, located in the open desert, constituting 35% of the microbiome.

We found no nodules on any of the root systems from the mature Ghaf trees that we excavated, but it would be impossible to say for certain that there were no nodules anywhere in these root systems. However, *P. cineraria* trees growing in desert farms with less abiotic stress produce abundant root nodules, similar to other Caesalpinioideae plants (24). The ultrastructure of root nodules showed intracellular bacteroids in some root cortical cells. These root nodules have 18% and 21% Actinobacteria on the nodule surface and nodule endosphere, respectively. *P. cineraria* plants growing in the growth chamber under optimal conditions produced the healthiest nodules, with 2.6% and 0.5% Actinobacteria in the NSM and NEM compartments, respectively, one indication of the very different microbial communities that develop in a growth chamber compared to those in a farm environment. Some actinobacterial isolates purified from *P. cineraria* in our study have a transcriptionally active ACC deaminase gene, which is known to confer drought tolerance in several bacteria (25, 26). OTU14 (*Actinophytocola* sp.) is exclusively present in the roots but absent in the root nodules. *nif*H gene fragments of *Actinophytocola* sp. were also detected in the root metagenomes and metatranscriptomes of *C. mopane* trees (27). It is well documented that Actinobacteria are more abundant in the root microbiomes of plants growing in limited nutrients, salt stress, water stress, and heat stress (28–31). Many actinobacterial members confer abiotic stress tolerance to many plant species (29, 32). It is also known that some Actinobacteria can produce nitrogenase enzymes and help some plants with associative nitrogen fixation (33). Additional studies may help determine Actinobacteria’s influence on root nodulation and nitrogen fixation in desert environments.

The root nodules of farm trees and growth chamber-grown plants were examined using 16S rRNA gene amplicon analysis, revealing that the *Ensifer* genus was the predominant microbial group. However, when analyzing the *nif*H amplicons, it was observed that *Ensifer* was the second most dominant genus, followed by *Herbaspirillum*. These findings suggest that not all the *Ensifer* isolates in the root nodules of *P. cineraria* possess nitrogenase gene clusters. *Ensifer* is among the dominant genera present in the root nodules of certain other *Prosopis* species, as well as other legumes such as soybean and various other legumes (34–37).

Shotgun metagenome and metatranscriptome data offer higher taxonomic resolution, cover all genetic information, and provide better quantitative insights compared to amplicon sequencing (38, 39) and were useful for assembly and binning, metabolic function profiling of all the genes, including the genes related to nitrogen fixation. Our analyses showed that the host plant DNA constitutes 2.2%–11.4% of the total nodule DNA, whereas the host plant RNA accounts for 88%–96% of the total nodule metatranscriptome. Hence, the plant DNA is massively over-expressed compared to the prokaryotic DNA in the nodules. When one takes into account the very different gene densities in bacteria (~1 gene/kb) versus *P. cineraria* (~1 gene/20 kb), then it is clear that the average *P. cineraria* genetic expression is ~170-fold stronger than the bacterial gene expression, on a per gene basis. One expects that most of these plant gene expression activities, which are enriched in the following paragraphs and supplementary material, are used to provide services to the nodule bacteria, thus indicating the huge energy commitment that this desert tree provides to acquire fixed nitrogen from the low fertility soils that it inhabits.

Several bacterial genes were found to be more abundant in the metagenomes. qPCR analysis showed that the *nif*H genes from *Ensifer* sp. are highly abundant (over 20,000-fold) in *P. cineraria* root nodules compared to *nif*H genes from other species. During the interaction of legume plant roots with rhizobia, the plant-derived infection threads transmit bacteria into the cortical cells. The bacterium grows and divides in the infection thread (40). When bacteria are released from infection threads, they become enclosed in a plant-derived membrane and differentiate into bacteroids, which are endosymbiotic, morphologically distinct, nitrogen-fixing symbionts. It was shown in the *Sinorhizobium meliloti:Medicago sativa* interaction that bacteroids in symbiotic root nodules have a higher DNA content than free-living cells (41). After infection, the host cells enlarge by cell expansion, amplifying the genome via endoreduplication cycles. The mature nitrogen-fixing symbiotic cells are large and contain several tens of thousands of symbiosomes with multiple bacteroids. In the indeterminate nodules, characterized in *Medicago*, *Pisum*, *Vicia*, etc., the symbiotic nodule cells enlarge gradually, consistent with numerous endoreduplication cycles, reaching ploidy levels of 32C and 64C, and are ~80-fold larger than the diploid nodule meristematic cells (42–44).

Metatranscriptome analysis allows the study of global transcription patterns of a host and its microbiomes in the root nodules with a single RNA preparation, thus providing internal consistency and broader scope to understand the functional contribution of each partner. For a rhizobial isolate such as *Sinorhizobium meliloti*, the shift from a free‐living saprophytic to an intracellular symbiotic lifestyle involves shutting down many basic metabolic processes on the chromosome, including nitrate assimilation, growth, and cell division, while switching on genes for micro‐aerobic respiration and N fixation on the symbiotic plasmids (45). Several plant genes related to carbohydrate metabolism (e.g., pyruvate kinase, decarboxylase, phosphoenolpyruvate carboxylase (PEPC), and respiration (e.g., cytochrome c oxidase) are highly expressed in the nodule metatranscriptomes. PEPC can comprise up to 2% of the soluble protein in root nodules of alfalfa (46). Alfalfa nodule PEPC is constitutively expressed in low levels in roots. In nodules, expression of PEPC polypeptide increases several-fold, resulting in increased PEPC activity (47). In addition, a strong interdependence of PEPC and nitrogenase activity was reported (48). PEPC-mediated CO_2_ fixation in nodules results in the synthesis of C4 dicarboxylic acids, viz., aspartate, malate, fumarate, etc., which can be transported into bacteroids with the intervention of dicarboxylate transporter protein. Evidence that dicarboxylic acids are the main respiratory substrates of bacteroids has been reported (49–51). The differentially expressed plant genes resulted in an increased turnover of some metabolic pathways. For example, tryptophan metabolism was found to be enhanced in the root nodules. Tryptophan is the precursor of auxin biosynthesis. It was shown that root nodules have high levels of auxins compared to surrounding tissue (52). Apigenin to luteolin pathway is significantly enhanced in nodule metatranscriptomes. Luteolin was known to induce expression of nodulation genes (nod ABC) in *Rhizobium meliloti* (53).

In summary, field and growth chamber results provided an overview of the total diversity and functional diversity of nodule microbiome, including the diazotroph population, associated with the root nodules of *Prosopis cineraria*. Our analysis has shown that the nodules have diverse microbial taxa, of which only a few perform nodulation and/or nitrogen fixation functions. The metagenomic and metatranscriptomic analyses, supported by control inoculation studies with pure isolates, showed that *Ensifer*/*Sinorhizobium* is the most important genus that fixes nitrogen in *P. cineraria*. However, several other microbial members, such as *Rhizobium* sp., *Agrobacterium* sp., and *Dechloromonas* sp., have transcriptionally active nitrogen-fixing genes in the nodules. Though the nodules were swamped by bacterial DNA, very few bacteria and pathways were found to be transcriptionally active in the nodules. In fact, the plant transcriptome is much more dominant than the bacterial transcriptome, suggesting that more plant genes play active roles in the nodulation, nitrogen fixation, nodule nutrient provision, and nitrogen transport activities than bacterial genes. Water stress, which normally affects plant growth and photosynthesis rates, also had a profound negative effect on nodulation under controlled conditions. This study suggests that this may be one of the reasons for the absence of detected nodules in the *P. cineraria* trees growing in the open desert. Actinobacteria members, which may confer drought tolerance but should not affect nodulation efficiency, may be immensely helpful in the greening of arid lands. The diazotrophs found to be transcriptionally active in the healthy nodules are also excellent candidates for soil improvement, which can be tried in mixed inoculations along with *Ensifer* sp. to improve nodulation and nitrogen fixation in desert environments.

## MATERIALS AND METHODS

This study pursued a comprehensive approach to collect, process, and analyze samples from *P. cineraria* in natural and controlled environments.

### Sampling of the soil, rhizosphere, and roots for microbiome analysis

We collected *P. cineraria* samples from three mature *P. cineraria* trees, aged over 10 years, located in the desert region near Al-Ain, UAE, during November 2017. The root samples were collected by carefully excavating up to 0.5M depth. The tertiary roots were collected at about 0.5M depth, and the root samples were stored on ice and processed the next day in the lab. One tree root system was excavated to a depth of 3M, and the roots were sampled at 0.5M, 1M, and 1.5M (Fig. S1A). Because the soil was sandy and dry, very little or no soil stuck to the roots. We collected sand next to the roots, which constituted the rhizosphere soil (Rh) fraction. The bulk soil (BULK) samples were collected at least 10M away from the root canopy.

We also collected *P. cineraria* root samples from a polyhouse tunnel (desert farm) (Fig. S1B) covered with a plastic sheet to control light and heat affecting moisture retention, and the trees were irrigated regularly. A total of three trees, over 3 years old, were sampled. The root nodules were sampled from the roots above 0.5M depth. The collected nodules were stored on ice and processed the next day in the laboratory.

To assess nodule microbiome diversity under controlled conditions in the growth chamber, surface-sterilized seeds of *P. cineraria* seedlings were grown in 8 × 8 cm pots with a 1:1 mixture of soil collected from the rhizosphere of a tree growing in the desert and sterile potting soil. The potting soil was sterilized by autoclaving at 121°C for 30 min. Containers with only a sterile potting mix were used as a negative control. Dehulled *P. cineraria* seeds were surface sterilized by treating them for 1 min in 100% ethanol, 15 min in 30% commercial bleach, and six washes with sterile Milli-Q water. The seeds were then incubated in 50 mL of filter-sterilized ½ MS media without vitamins (Sigma Aldrich, USA) (pH 7.8) in a 250-mL flask with shaking at 250 rpm for 2–4 h at 37°C and finally rinsed with sterile Milli-Q water. Four seeds were planted in each pot, and the containers were maintained in saturated water in the greenhouse at 24°C, 65% humidity with an 18-h/8 h light/dark cycle. Germinated seedlings were harvested after 8 weeks. The soil was removed by washing from the roots, and seedlings were photographed. Nodules from the seedling roots were randomly selected (unless otherwise noted) to isolate microbes.

### Sample processing, metagenomic DNA, and RNA preparation

Each root and adherent soil sample from the desert trees were separated into two fractions. The first was a rhizosphere fraction (Rh), which was defined as the soil that washed off the root. The second was the root’s ectosphere (rhizoplane) plus endosphere (REE), which was the total washed root, as described in our previous study (12). For nodules, each nodule, after gentle rinsing with sterile water, was transferred to a 15-mL falcon tube with 1 mL phosphate buffer (pH 8.0), 10 µL lysozyme (100 mg/mL), 0.1 U chitinase, 1 µL RNAse A (100 mg/mL), 0.1 g sterile 710–1,180 micron beads, and 0.2 g sterile 212–300 micron glass beads. This tube was shaken at 250 rpm for 30 min at 37°C (12). The resultant enzyme solution was centrifuged at high speed to collect microbial fractions that constitute the nodule surface microbiome. The nodules rinsed with sterile water three times after this step are enriched in endophytic microorganisms, which we refer to as the nodule endophytic microbiome.

Total DNA was extracted from the bulk and rhizosphere samples using the Qiagen Soil DNA Extraction Kit (Qiagen, USA). DNA from the NSM fraction was isolated using the Quick-DNA Fungal/Bacterial Miniprep Kit (Zymoresearch, USA). Total DNA was extracted from the root and nodule samples using the DNeasy Plant Mini Kit (Qiagen, MD, USA). The total RNAs from root and nodule samples were prepared using a modification of a previously published method (54).

### Illumina amplicon sequencing and data analysis

16S and nifH amplicon libraries from the roots and nodules were prepared by using V3-V4 sequence-specific primers as previously described (12, 55). Sequence data sets were trimmed, clustered, and classified in MOTHUR (56) as described in our previously study (12). In contrast, *nif*H amplicon reads were processed, and *nif*H OTUs were clustered as reported in our previous study (55). The OTUs were further analyzed after retaining OTUs occurring >3 times in the whole data set. We further processed the samples to focus on abundant reads by sub-sampling 10,000 reads from each sample using the subsample function in MOTHUR. The taxonomy-based classification of *nif*H gene sequences was obtained from the database created from the sequences in the NCBI database and the RDP Fungen database (57).

### Shotgun metagenomic library preparation and analysis

Metagenomic DNA from the root nodules of six independent 8-week-old *P. cineraria* seedlings growing in the growth chambers, as described previously, was used to prepare Illumina shotgun genomic DNA libraries using the Truseq DNA Library Prep Kit (Illumina, USA). Illumina Nextseq PE150 sequencing was done at the Georgia Genomics and Bioinformatics Core, Athens, GA. Shotgun metagenomic data were analyzed with the MG-RAST pipeline 4.0.2 (58), where paired ends were joined, annotated, and binned. Sequence reads were annotated with the M5NR and RefSeq databases for taxonomic identification. The Subsystems database was queried for assistance in function identification, using a maximum *e*-value of 1e−5, 60% minimum identity, and 15 amino-acid minimum alignment length. Representative hit classification was used to quantify taxonomic abundance (58).

### Preparation of RNAseq libraries, Illumina sequencing, and analysis

Root RNAs and nodule RNAs from three plants from each of the two genotypes (DT and FT) were used for metatranscriptome analysis. Ribodepletion libraries were prepared by removing rRNA using Ribo-Zero rRNA Removal Kit (Bacteria) (Illumina, USA). Illumina PE150 sequencing on all these RNAseq libraries was performed at Macrogen, South Korea. These metatranscriptomes were analyzed with MG-RAST pipeline 4.0.2.

### Transcript assembly, expression, and pathway analysis of RNAseq data

We followed previously described procedures and scripts (59, 60) to analyze RNAseq data from three replicates of root samples and three nodule samples. The genome assembly of *Prosopis cineraria* (19) was used as the reference to guide the transcript assembling from cleaned reads with HiSAT2 (version 2.0.5) and StringTie (version 1.3.3b) (61). We used the same annotation methods described in our previous publication (62). More details on the RNAseq data analysis and annotation were provided in the supplementary information.

The methods for the purification and identification of rhizobial isolates from *P. cineraria* root nodules and control inoculation studies were described in the supplemental information.

## Data Availability

The data generated during this study have been submitted to NCBI-SRA database under the BioProject id: PRJNA967649.

## SUPPLEMENTAL MATERIAL

Data Set S1 spectrum.03617-23-s0001.xlsx Differentially expressed plant genes in the root nodules and *P* values.

Data Set S2 spectrum.03617-23-s0002.xlsx Significantly enriched pathways in the root nodules.

Supplemental figures spectrum.03617-23-s0003.pdf Fig. S1-S16.

Supplemental material spectrum.03617-23-s0004.docx Supplemental Methods, Results, and Discussion.

Table S1 spectrum.03617-23-s0005.xlsx The 16S bacterial OTUs from the bulk soil, rhizosphere soil, and root external and endophytic compartments, which are significantly different between three underground environments of *P. cineraria* trees growing in the desert.

Table S2 spectrum.03617-23-s0006.xlsx The 16S bacterial OTUs, which are significantly different between five underground environments of *P. cineraria* trees growing in the desert and the farm: the bulk soil, rhizosphere soil, root external and endophytic compartments, nodule surface, and nodule endosphere.

Table S3 spectrum.03617-23-s0007.xlsx Relative abundance of nitrogen metabolism related bacterial functions in the metagenomes and metatranscriptomes of *P. cineraria* root nodules as revealed by MG-RAST subsystems analysis.

Table S4 spectrum.03617-23-s0008.xlsx Comparison of gene copy number in the genomes and transcriptomes of *P. cineraria* root nodules.

Table S5 spectrum.03617-23-s0009.xlsx Metabolic pathways which are significantly enriched in the root nodules of *Prosopis cineraria*.

Table S6 spectrum.03617-23-s0010.docx qPCR primer list.

ASM does not own the copyrights to Supplemental Material that may be linked to, or accessed through, an article. The authors have granted ASM a non-exclusive, world-wide license to publish the Supplemental Material files. Please contact the corresponding author directly for reuse.
