# Supplementary material for: Microbiome properties in the root nodules of Prosopis cineraria, a leguminous desert tree
Source: Microbiol Spectr. 2024 Apr 16;12(6):e03617-23. doi: 10.1128/spectrum.03617-23 (PMC11237379; doi:10.1128/spectrum.03617-23)
Supplement: Supplemental figures — Fig. S1-S16. [file spectrum.03617-23-s0003.pdf]

SUPPLEMENTARY FIGURES

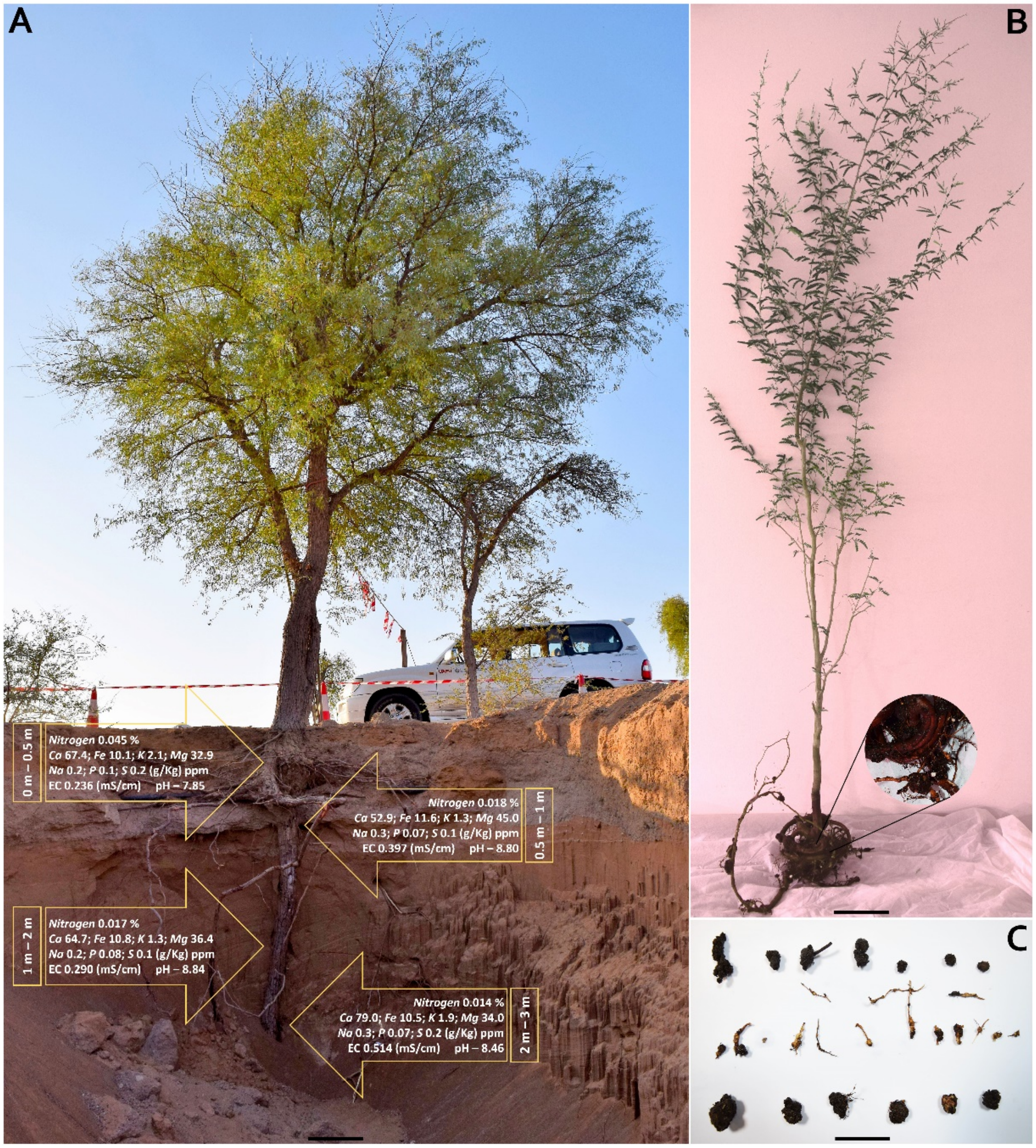

Fig S1. A) The soil where a *P. cineraria* tree is growing in the Al Ain desert was excavated down to 3 M for the current study. Root morphology and the mineral composition of rhizosphere soil at various depths are shown. B) A three-year-old *P. cineraria* tree grown in the desert farm was uprooted for the current study. The root nodules isolated for the analysis are shown. C) Individual indeterminate nodules separated from the roots of a three-year-old *P. cineraria*.

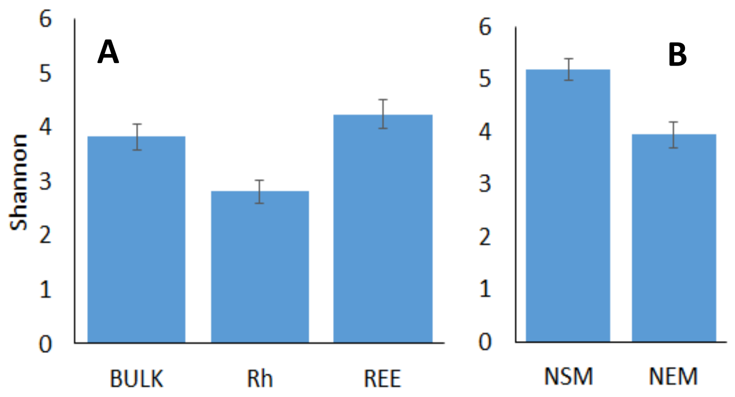

Fig S2. 16S bacterial diversity, as expressed by Shannon diversity index, in the soil, roots (A) and nodules (B) of *P. cineraria* trees growing in the open desert

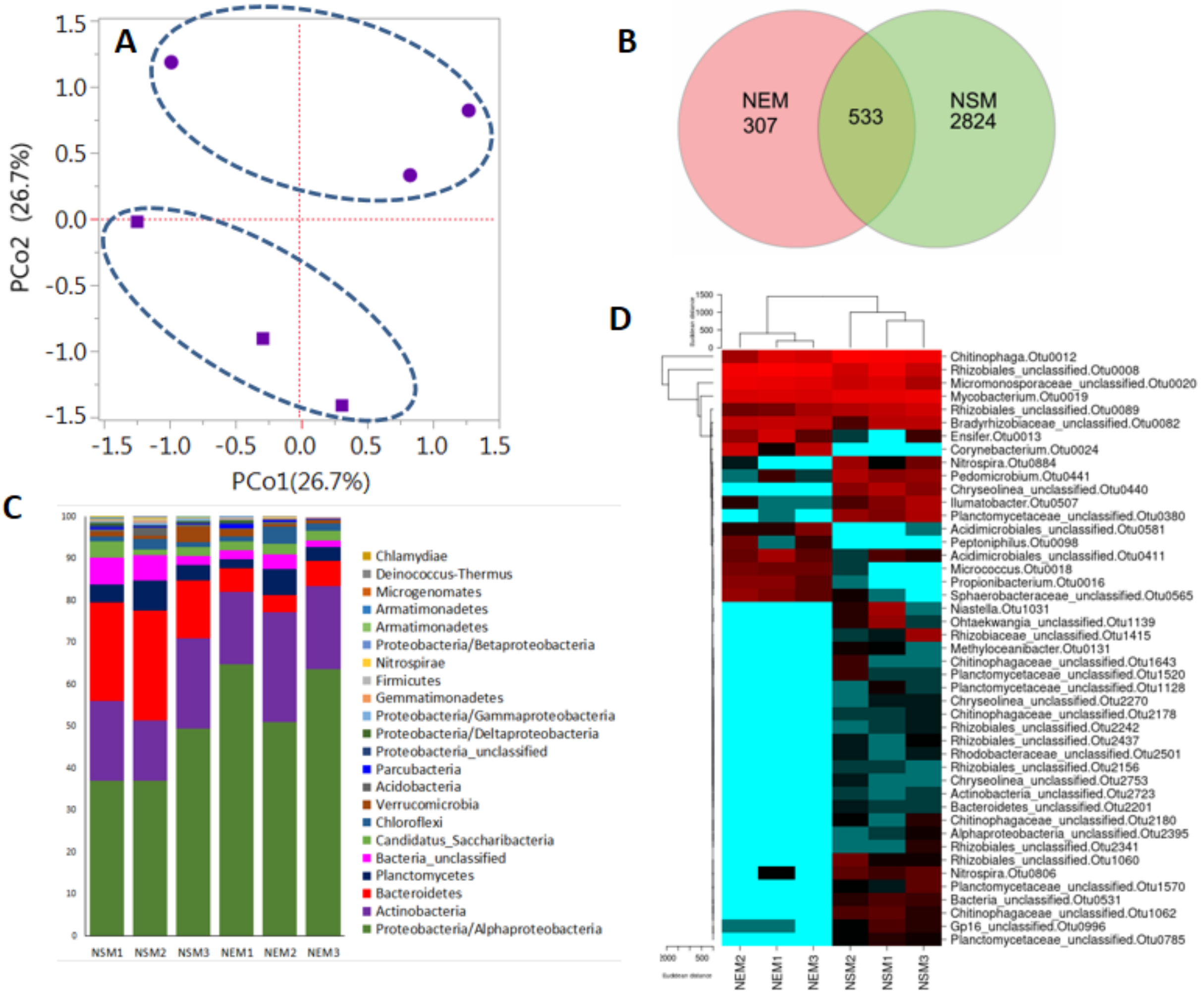

Fig S3. Bacterial diversity in the root nodules of *P. cineraria* trees growing in the desert farm. A) Principal Coordinates Analysis of 16S bacterial OTUs from the root nodules of *Prosopis cineraria* growing in the desert under irrigation. PCoA plots were generated from a distance matrix for the Jaccard indices set using the rarified 16S OTU table. Plots show the first two principal axes. Nodule surface microbiome (NSM) was shown in purple squares, and nodule endophytic microbiome (NEM) samples are shown in purple circles. B) Venn diagrams showing shared and unique OTUs from NSM and NEM. C) Relative abundance of bacterial phyla in the NSM and NEM compartments. The averages of each phylum for each sample of NSM and NEM were shown as six bars. D) A hierarchical cluster analysis of relative OTU abundance (Y-axis) of significant OTUs versus sample groups NSM and NEM used in the current study. Red indicates high abundance and blue indicates low abundance.

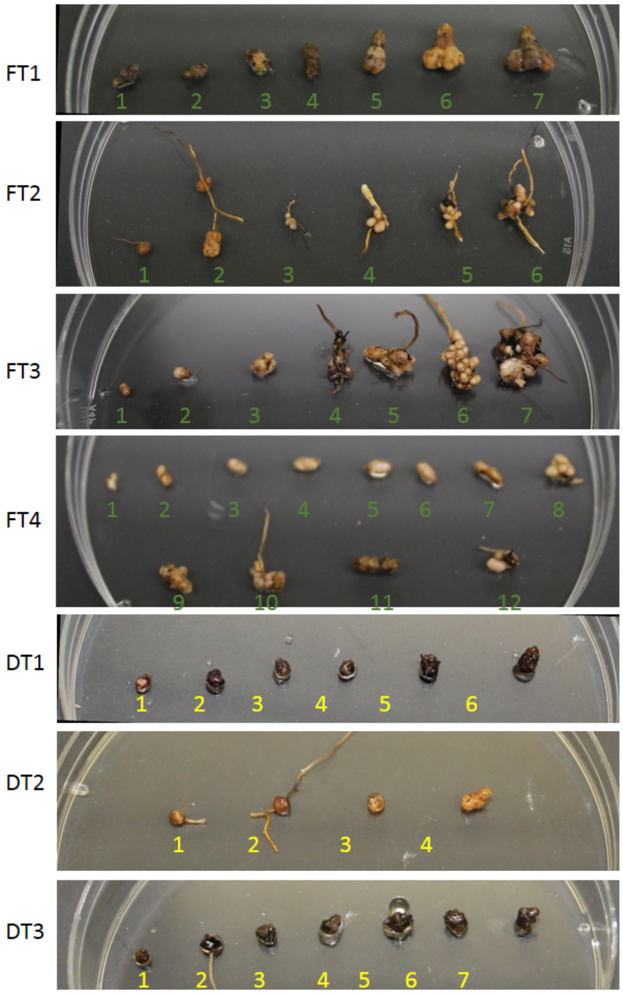

Fig S4. Morphology of root nodules harvested from *P. cineraria* seedlings grown in the growth chamber under controlled conditions of light, temperature, humidity and moisture. The seed from two *P. cineraria* genotype were collected from trees growing in the open desert (DT) and desert farm (FT). Nodules of different sizes were collected from the growth chamber-grown plants raised from four FT seed and three DT seed.

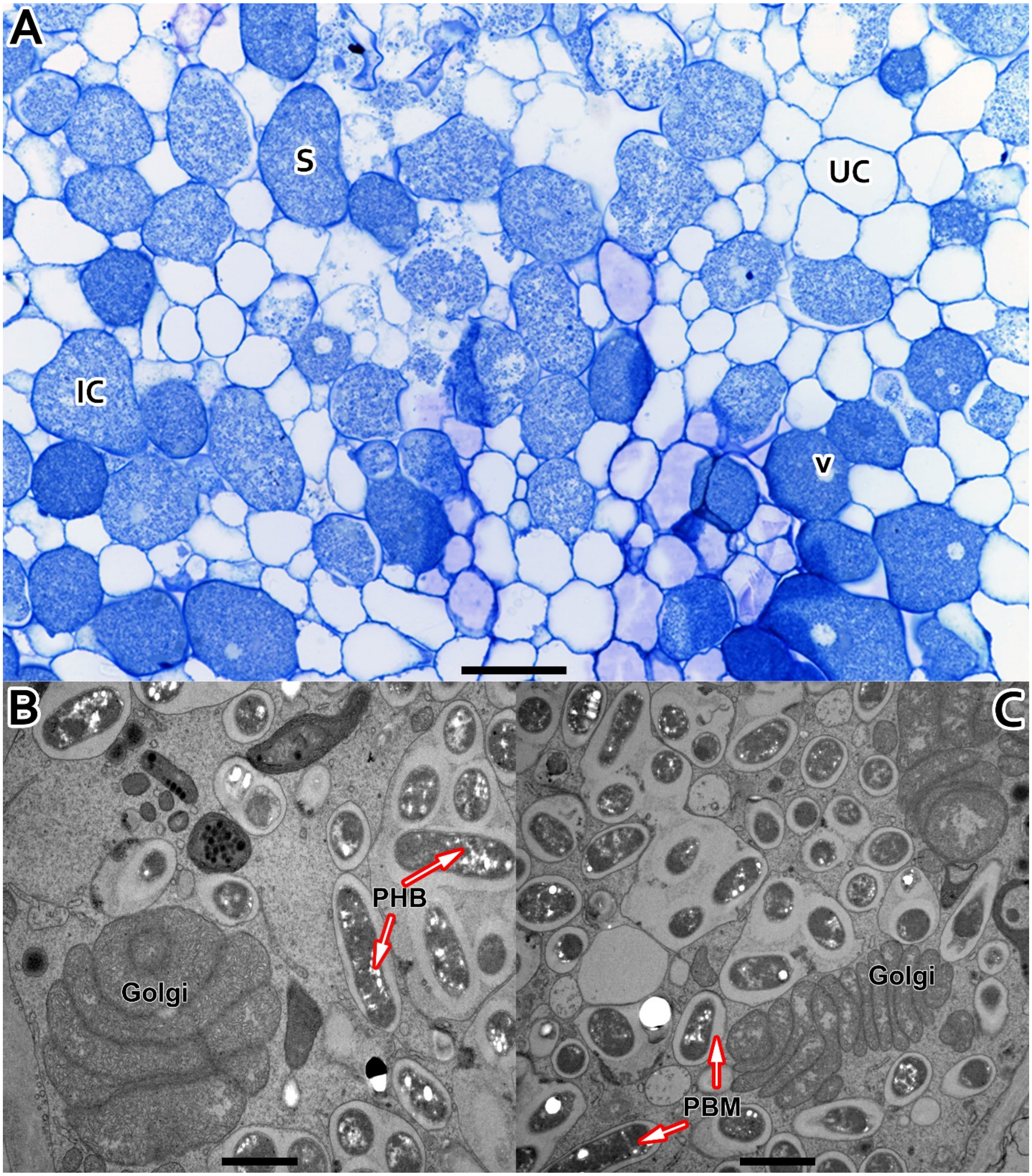

Fig S5. Phase-contrast and electron microscopy of *P. cineraria* root nodules. A) Cortical cells of the root nodules filled with bacteroids are shown. B) Bacteroids in the intracellular compartments are shown. Infected cells (IC), uninfected cells (UC), vacuole of infected cell (V), symbiosome (S). Examples of peribacteroid membrane (PBM) and poly-b-hydroxybutyric acid (PHB) energy storage particles are pointed out in the figures.

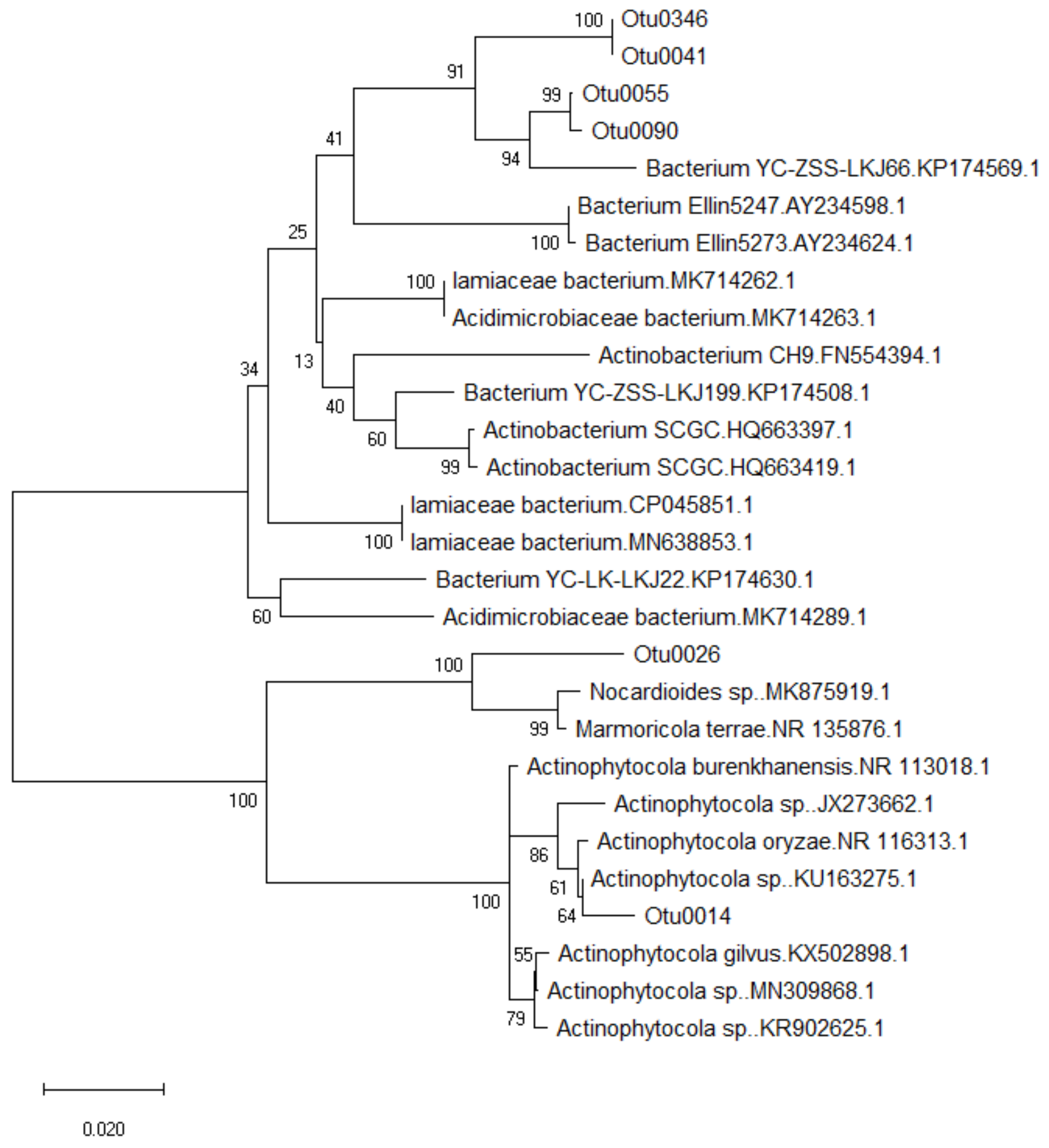

Fig S6. Evolutionary relationships of actinobacterial OTUs significantly enriched in the roots of *Prosopis cineraria* trees growing in the deserts and their nearest neighbors from GenBank. The evolutionary history was inferred using the Minimum Evolution method. The optimal tree with the sum of branch length = 0.55477040 is shown. The percentage of replicate trees in which the associated taxa clustered together in the bootstrap test (500 replicates) are shown next to the branches. The tree is drawn to scale, with branch lengths in the same units as those of the evolutionary distances used to infer the phylogenetic tree. The evolutionary distances were computed using the Maximum Composite Likelihood method and are in the units of the number of base substitutions per site. The ME tree was searched using the Close-Neighbor-

Interchange (CNI) algorithm at a search level of 1. The Neighbor-joining algorithm was used to generate the initial tree. The analysis involved 28 nucleotide sequences. All ambiguous positions were removed for each sequence pair. There were a total of 379 positions in the final dataset. Evolutionary analyses were conducted in MEGA X.

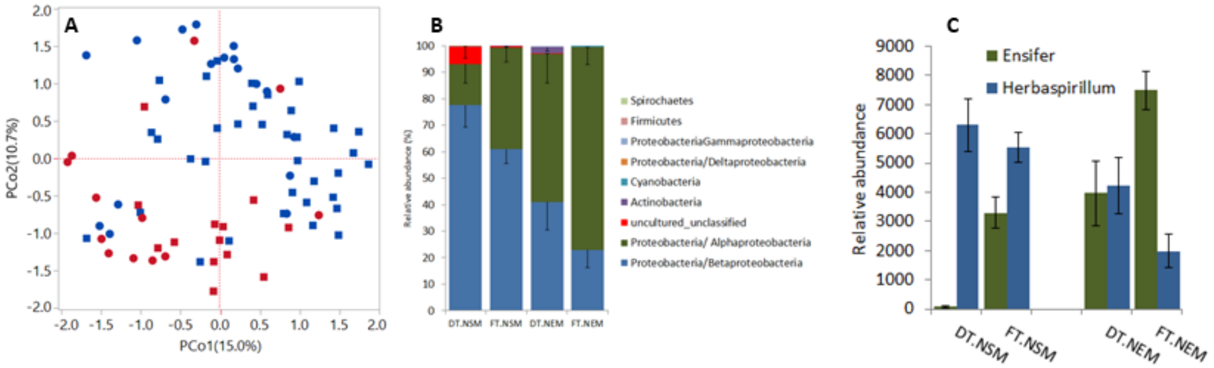

Fig S7. *Nif*H bacterial diversity in the root nodules of the growth chamber-grown *P. cineraria*. A) Principal Coordinates Analysis of *nif*H bacterial OTUs (NOTUs) from the root nodules of *Prosopis cineraria* growing in the growth chambers. PCoA plots were generated from a distance matrix for the Jaccard indices set using the rarified NOTU table. Plots show the first two principal axes. Nodules from DT tree seedlings are shown in red and FT tree seedlings are shown in blue. Nodule surface microbiome (NSM) were shown in squares, and nodule endophytic microbiome (NEM) samples are shown in circles. B) The relative abundance of *nif*H bacterial phyla in the NSM and NEM compartments of root nodules from DT seedling and FT seedlings. C) The relative abundance of genera *Ensifer* and *Herbaspirillum* in the NSM and NEM nodule compartments of DT and FT seedlings

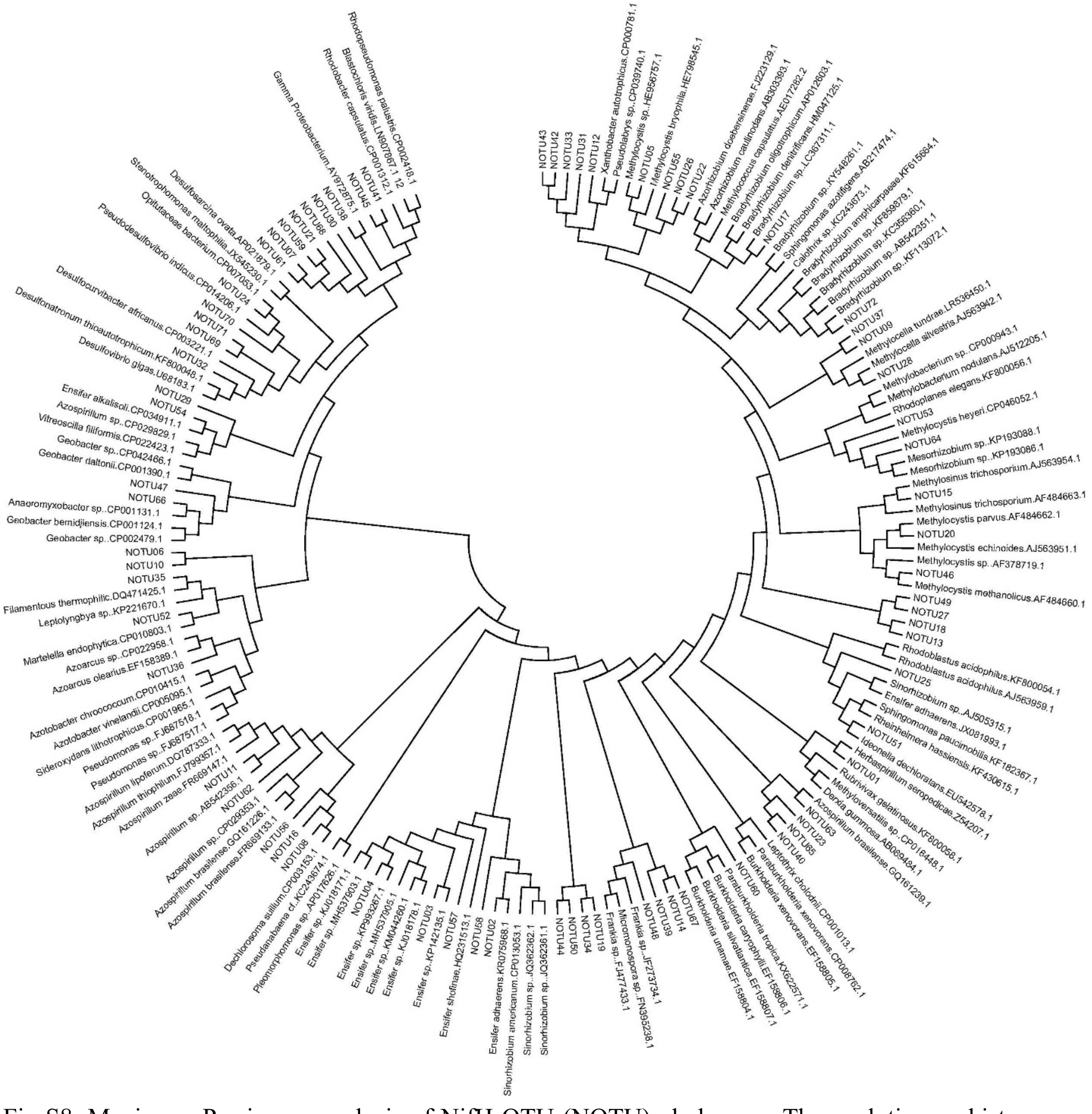

Fig S8. Maximum Parsimony analysis of NifH OTU (NOTU) phylogeny: The evolutionary history was inferred using the Maximum Parsimony method. The bootstrap consensus tree inferred from 100 replicates is taken to represent the evolutionary history of the taxa analyzed. Branches corresponding to partitions reproduced in less than 50% bootstrap replicates are collapsed. The MP tree was obtained using the Subtree-Pruning-Regrafting (SPR) algorithm with search level 1 in which the initial trees were obtained by the random addition of sequences (10 replicates). The analysis involved 177 nucleotide sequences. All positions with less than 100% site coverage were eliminated. That is, fewer than 0% alignment gaps, missing data, and ambiguous bases were allowed at any position. There were a total of 287 positions in the final dataset. Evolutionary analyses were conducted in MEGA5.

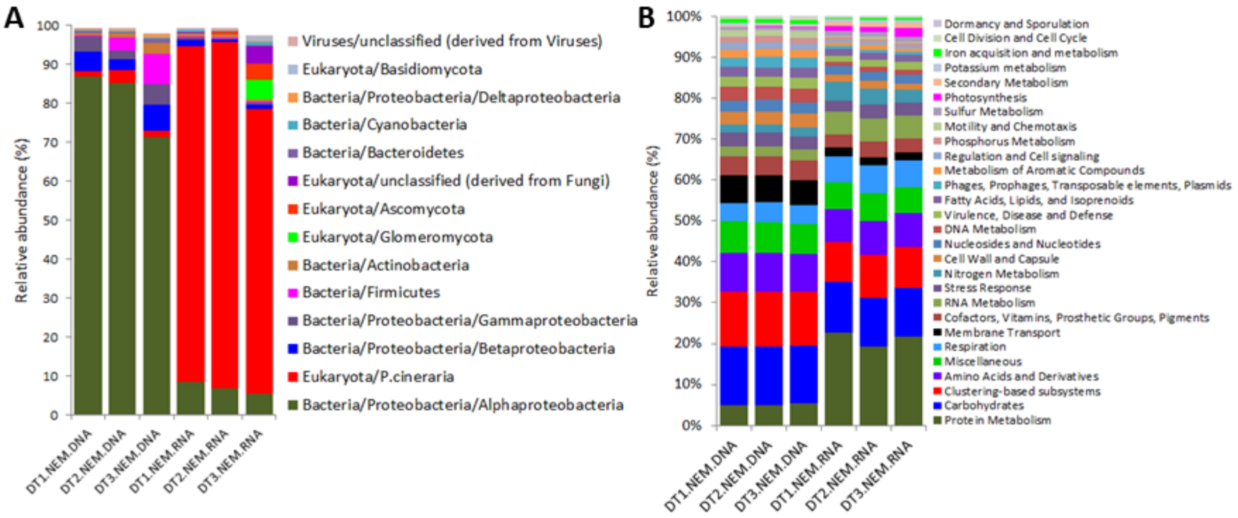

Fig S9. Comparative analysis of metagenomes and metatranscriptomes. A) The relative abundance of host and microbial phyla inhabiting *P. cineraria* root nodules. The Proteobacteria phylum was further divided into classes (Alpha-, Beta-, Gamma-, Deltaproteobacteria) and their relative abundance is shown separately. Note the tiny quantity of *P. cineraria* DNA and the relatively vast amount of *P. cineraria* RNA (both in red). B) Relative abundance of metagenome and metatranscriptome functions in *P. cineraria* root nodules.

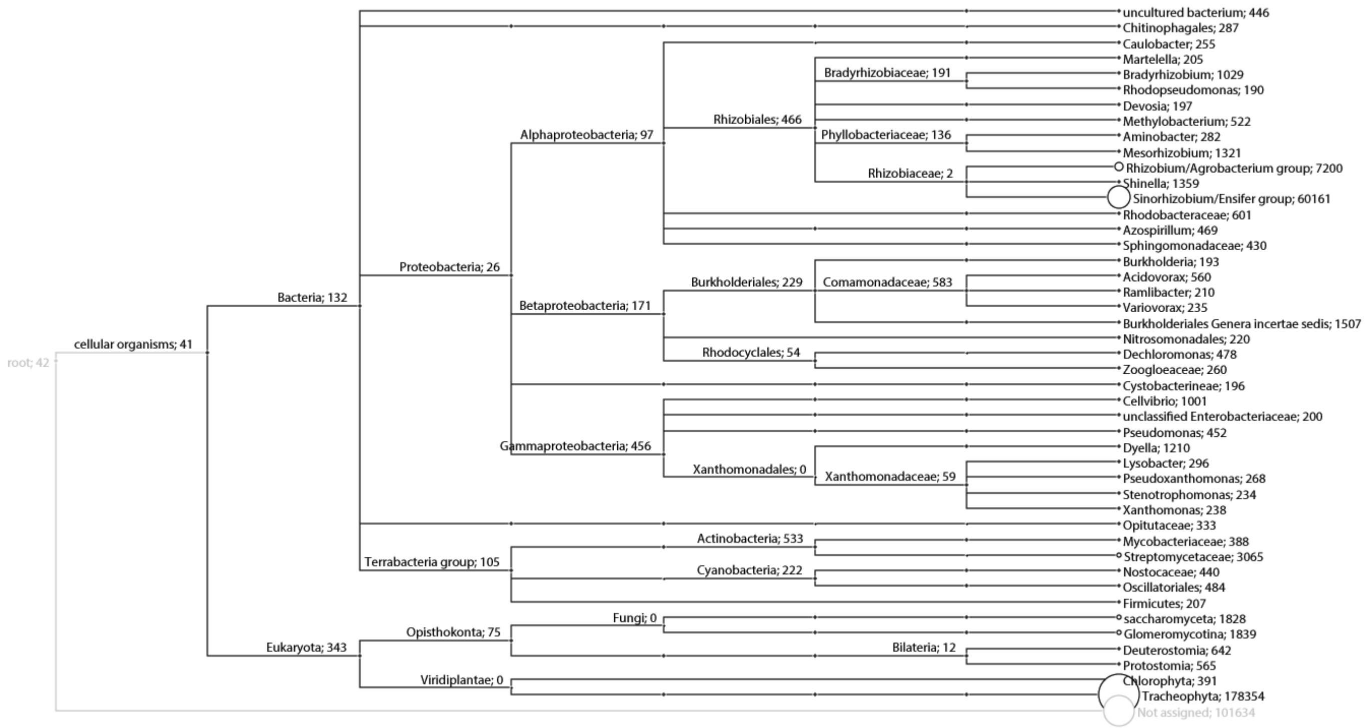

Fig S10. Phylogeny of active microbiome in the root nodule metatranscriptome of *P. cineraria.* MEGAN analysis was based on a BLASTN comparison of individual transcripts with NCBI-NR. Number of reads that are homologous to each taxon is shown after each semicolon.

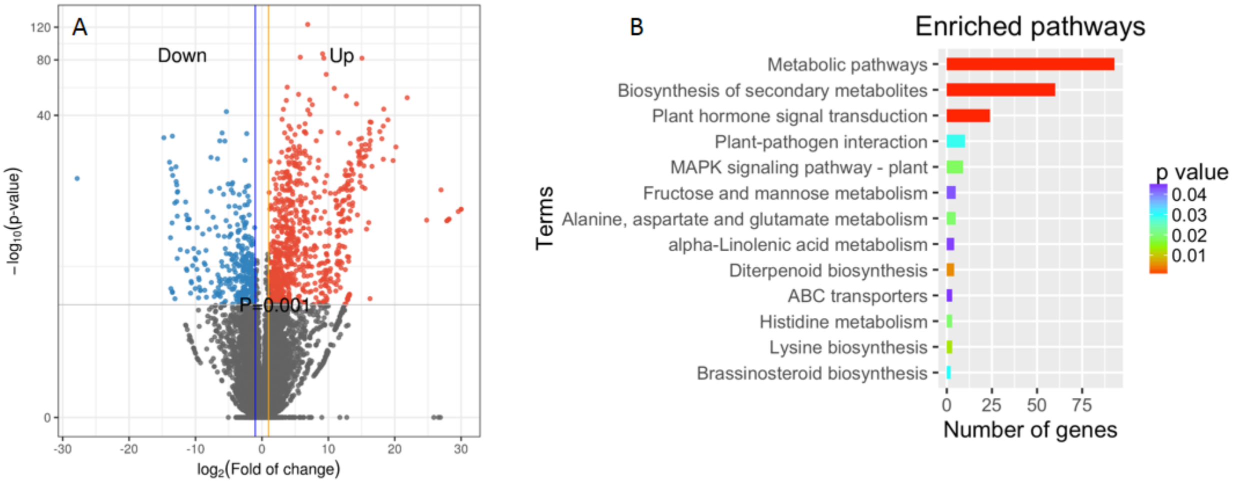

Fig S11. Differentially expressed genes (DEGs) in the root nodules of *P. cineraria* A) The distribution of up- and down-regulated genes in the root nodules. The differentially expressed genes (DEGs) were shown in color (up-regulated genes in red and down regulated genes in blue) with at least a two-fold change and p value < 0.001. (B) The enriched pathways with the number of DEGs.

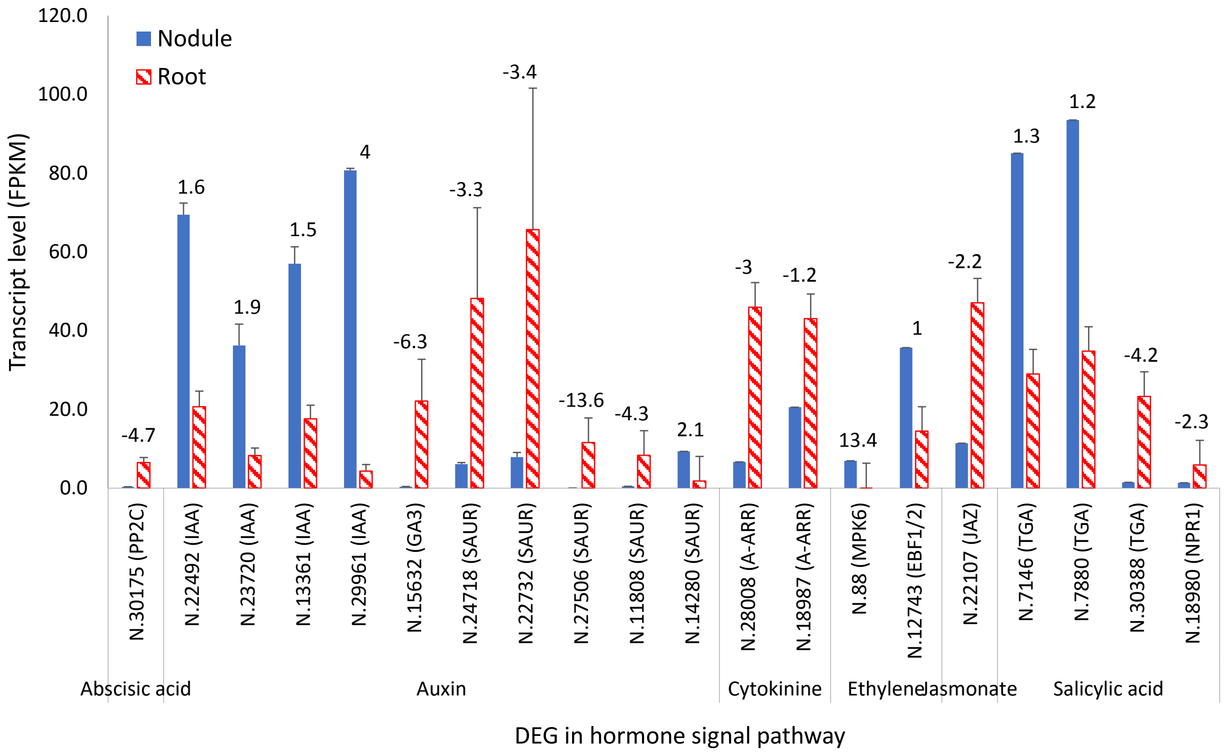

Fig S12. Transcript levels of selected differentially expressed genes (DEG) and their related pathways (x-axis) in the root nodules of *P. cineraria*. Whole list of differentially expressed genes are presented in the excel file “Supplementary data - Differentially expressed plant genes in the root nodules and p values”. The fasta sequences of The DEGs are provided in the “Supplementary data - *P. cineraria* root nodule DEG sequences P0.001.final”.

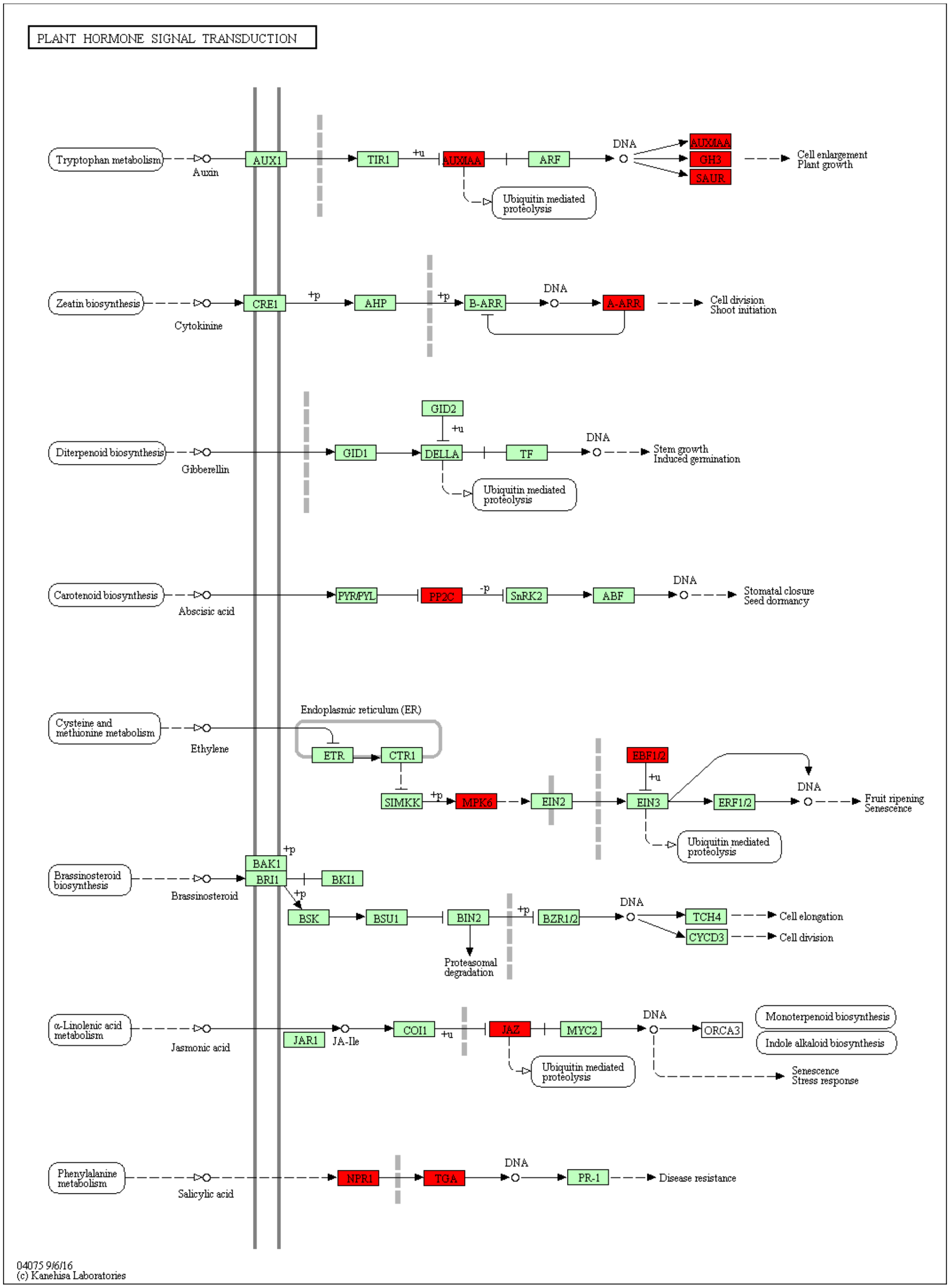

Fig S13. Hormone biosynthesis and metabolism pathways enriched in the nodules of *P. cineraria*. Hormone biosynthesis and metabolism pathways enriched in the nodules of *P. cineraria*. Red color indicates upregulated genes (positive fold value) and green color represents down-regulated (negative fold value) genes.

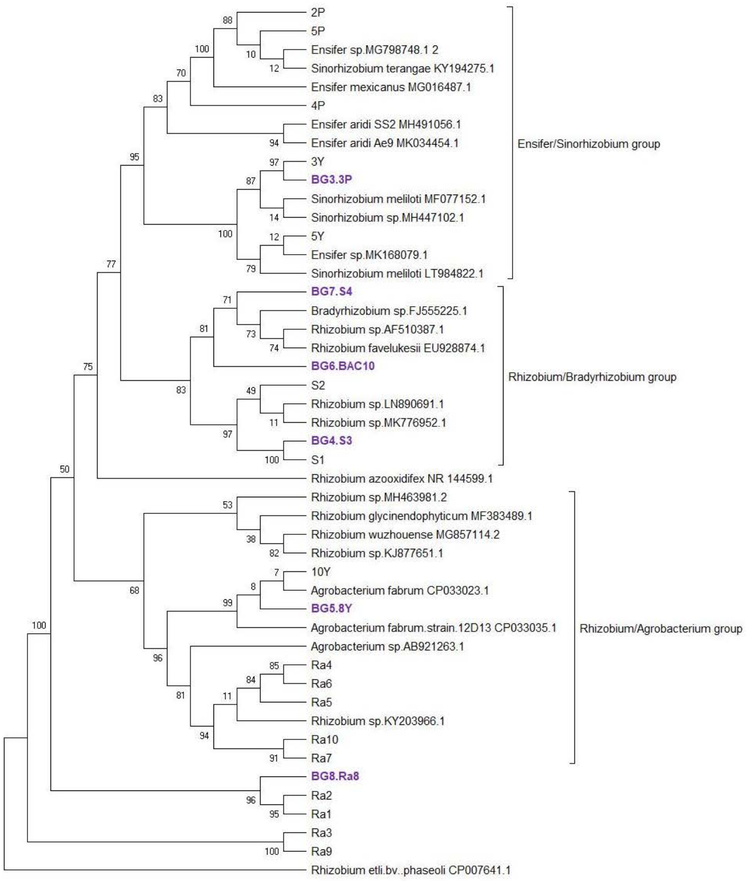

Fig S14. Maximum Parsimony analysis of 16S rRNA gene sequences of pure bacterial isolates from the root nodules of P. cineraria. The evolutionary history was inferred using the Maximum Parsimony method. Tree #1 out of 2 most parsimonious trees (length = 1216) is shown. The consistency index is (0.775691), the retention index is (0.914346), and the composite index is 0.761704 (0.709250) for all sites and parsimony-informative sites. The percentage of replicate trees in which the associated taxa clustered together in the bootstrap test (500 replicates) are shown next to the branches. The MP tree was obtained using the Subtree-Pruning-Regrafting (SPR) algorithm with search level 1 in which the initial trees were obtained by the random addition of sequences (10 replicates). The analysis involved 47 nucleotide sequences. There were a total of 2160 positions in the final dataset. Evolutionary analyses were conducted in MEGA X.

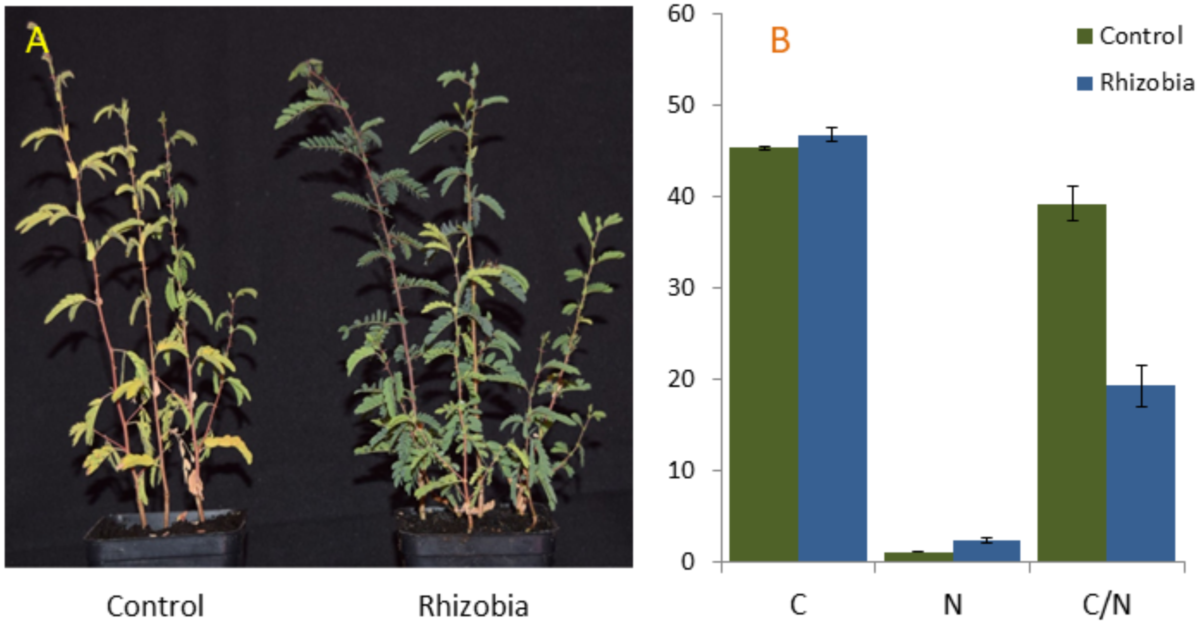

Fig S15. Nodulation efficiency of rhizobial strains BG3(*Ensifer meliloti*), *BG4 (Rhizobium sp. BG4), BG5(Agrobacterium fabrum), BG6 (Rhizobium sp. BG6), BG7(Rhizobium grahamii) , BG8(Sinorhizobium sp.*). Single inoculations of each individual strain and a mixture of all the six strains were tested.

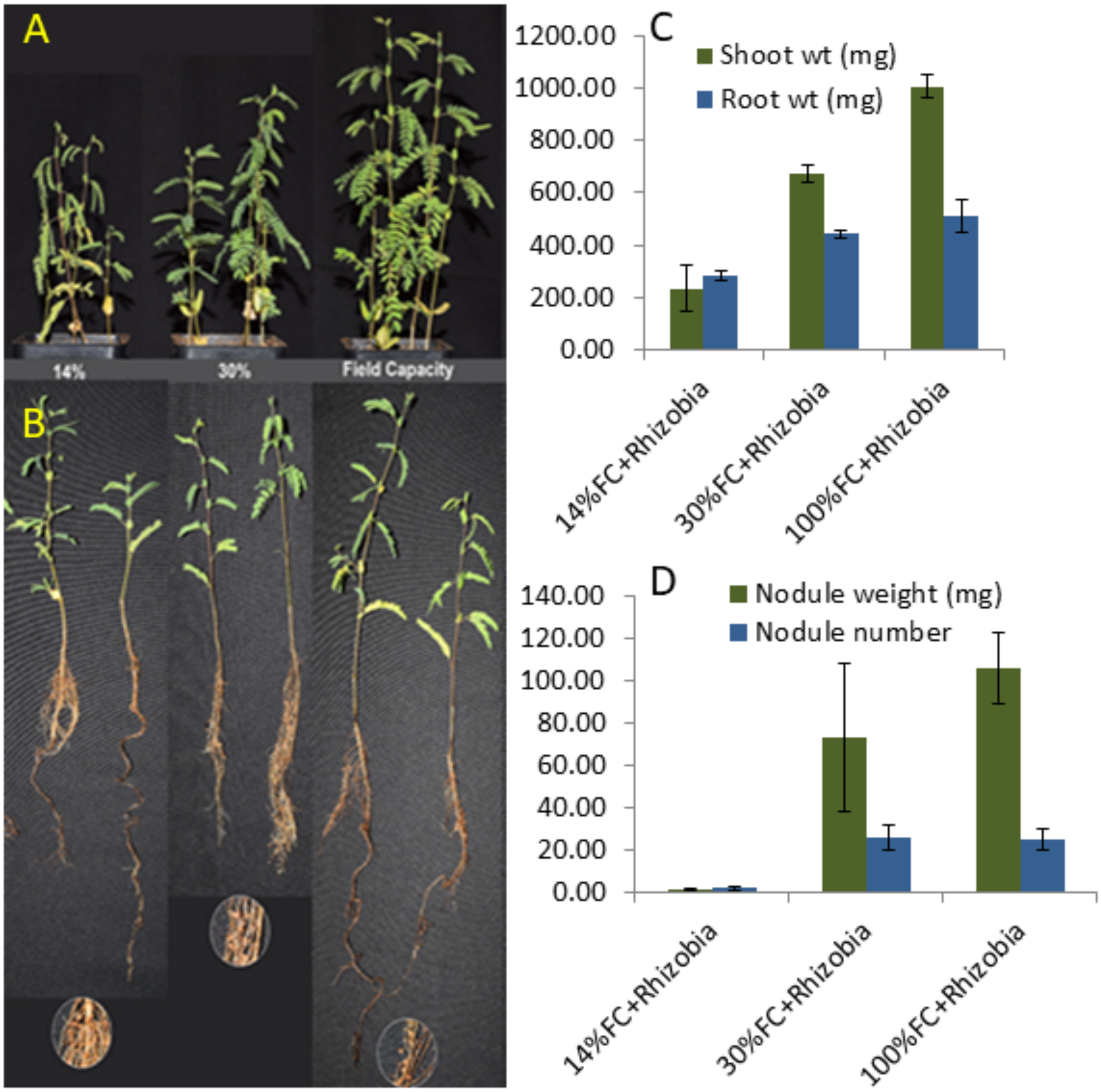

Fig S16. Effect of water stress on the root nodulation by rhizobia and plant growth. *P. Cineraria* seedling were grown in the presence of rhizobial mix for six weeks under water stress at different field capacities (FC) (14% and 30 % and 100% FC conditions. The phenotypes of the above ground (A) and below ground (B) parts of the plants are shown. Effect of shoot and root growth (C), nodule number and nodule weight (D) are shown. Error bars represents standard error between three biological replicates from independent pots. Asterisks indicate significant differences between water stress treatments.
