# Supplementary material for: Microbiome properties in the root nodules of Prosopis cineraria, a leguminous desert tree
Source: Microbiol Spectr. 2024 Apr 16;12(6):e03617-23. doi: 10.1128/spectrum.03617-23 (PMC11237379; doi:10.1128/spectrum.03617-23)
Supplement: Supplemental material — Supplemental Methods, Results, and Discussion. [file spectrum.03617-23-s0004.docx]

**Supplemental information**

**Supplementary methods**

### **Electron microscopy of nodule sections**

Mature nodules were collected from an actively growing *P. cineraria* seedling and cut into 2-3 mm thick slices using surgical blades. Slices were washed with 0.1M phosphate buffer at pH 7.2 (1) and then immersed in Karnovsky’s fixative at pH 7.2 (2) for 24 hours at 4^o^C. The fixative was discarded, and the tissue was rinsed with phosphate buffer. The tissues were then postfixed with 1% osmium tetroxide for 1hr. After washing with distilled water, slices were dehydrated in a series of graded ethanol solutions from 30% to 100% and finally in propylene oxide. Then, slices were infiltrated, embedded in Agar100 epoxy resin, and polymerized at 65^o^C for 24 hours. Semi-thin and ultrathin sections were cut with Reichert Ultracuts and ultramicrotome (C. Reichert AG, Austria). Semi-thin slices (130nm thickness) were mounted after staining with 1% aqueous toluidine blue. The images were captured using Nikon Eclipse LV 100 upright microscope with Ds-RI2 Camera and 20X and 50X objectives. The ultrathin sections were placed on an Electrothermal slide drying bench at 55oC. The ultra-thin sections (95nm) on 200 mesh Cu grids were contrasted with Uranyl Acetate (3). After three washes with water, the sections were treated with Lead Citrate (4) double stain. Grids were examined and photographed at different magnifications under the Philips CM10 Transmission Electron Microscope (Netherlands).

### **Sample processing, metagenomic DNA, and RNA preparation**

Each root and adherent soil sample from the desert trees was separated into two fractions. The first was a rhizosphere fraction (Rh) that constitutes the soil adjacent to the root, acquired by rinsing the root in sterile water. The second fraction contained the root’s ecto and endo-sphere microbes (REE). 2 g of REE fraction was transferred to a 15 ml falcon tube containing 5 ml of phosphate buffer (pH 8.0), 50 μl of lysozyme (100 mg/ml), 0.5 U of Chitinase, 5 μl of RNAseA (100 mg/ml), 2 g of sterile 710–1180-micron beads, and 2 g of sterile 212–300-micron glass beads. The tube was then shaken at 250 rpm for 30 minutes at 37°C and subsequently rinsed with sterile water. This enzyme-treated fraction is enriched with the root-endosphere microbiome (REN) [33]. Each nodule, after gentle rinsing with sterile water, were transferred to a 15 ml falcon tube with 1 ml phosphate buffer (pH 8.0), 10 μl lysozyme (100 mg/ml), 0.1 U Chitinase, 1 μl RNAseA (100 mg/ml), 0.1 g sterile 710–1180-micron beads, 0.2 g sterile 212–300-micron glass beads and were shaken at 250 rpm for 30 min @ 37°C (5). After removing and saving the enzyme solution, it was centrifuged at high speed to collect microbial fractions that constitute the Nodule Surface Microbiome (NSM). The nodules rinsed with sterile water three times after this step are enriched in endophytic microorganisms, which we refer to as the Nodule Endophytic Microbiome (NEM).

### **Purification of RNA, cDNA synthesis, and Real-Time PCR**

Total RNA was extracted from samples described above and treated with DNAase according to a protocol from “RNeasy® MinElute® Cleanup Handbook (Qiagen, USA). Subsequently, the DNAase treated samples were purified using QIAGEN “RNeasy® MinElute® Cleanup Kit (QIAGEN, USA).

The qPCR primers, listed in Table S6, were designed by Primer3 software in Geneious Prime 10.1. The RNA was isolated from the roots of three plants grown in the growth chamber without rhizobial inoculation and from the nodules of three plants growing in similar conditions with rhizobial inoculation as previously described. First-strand cDNA was synthesized from purified RNA samples using “QuantiTect® Reverse Transcription Kit (Qiagen, USA) using a random primer mix. The actin gene transcripts were used for normalizing the expression across all the samples. Real-time PCR was performed on the first strand cDNA reaction product in the StepOne Plus™ system (Applied Biosystems, USA) and using Fast SYBR™ Green Master Mix (Bio-Rad, USA). The genes chosen from the plant were enolase, GADP, NF-Y10A, and STP, which had an average of 254, 265, 163, and 37 fragments per million reads, respectively, in our ribodepletion RNAseq analysis.   At the same time, we also tested the expression of bacterial genes Ensifer nitrogenase alpha subunit, Ensifer nitrogenase beta subunit, and Mesorhizobium NifH, which represented 501, 599, and 21reads, respectively.

The *P. cineraria* glyceraldehyde-3-phosphate dehydrogenase (GAPD) was amplified in parallel with the target genes allowing gene expression normalization and quantifying. Three independent biological replicates of each sample and three technical replicates of each biological replicate were used for real-time PCR analysis. To determine relative fold differences for each gene in the nodule samples, the CT value for each target gene was normalized with the CT value of the reference gene. It was calculated relative to a calibrator (root samples) using the ∆∆CT method (6).

### **Transcript Assembly, Expression, and Pathway Analysis of RNAseq**

We followed previous described procedures and scripts (7, 8) to analyze RNA-Seq data from three replicates of root samples, and three nodule samples. The genome assembly of *Prosopis cineraria* (9) was used as the reference to guide the transcript assembling from cleaned reads with HiSAT2 (version 2.0.5) and StringTie (version 1.3.3b) (10). Transcripts were discarded if only supported by less than three RNA-Seq reads across all samples and less than 200 bp, and the remaining transcripts were used for subsequent analysis. The differential expressed genes and transcripts in nodule samples were identified using R package DEseq2 (version 1.16) (11) at a cutoff of at least a two-fold change and p-value < 0.001 using root samples as a control. For annotation, we used the same methods described in our previous publication (12). Briefly, transcripts were used to search using BLASTx with an E-value threshold of E-5 against the NR (NCBI non-redundant protein sequences), UniProtKB (Swiss-Prot and TrEMBL), KOG (euKaryotic Orthologous Groups) databases, and against the Pfam database by HMMER3 with an E-value of 1E-5. Based on annotation ID, gene ontology (GO) terms were retrieved from the Gene Ontology database (http://www.geneontology.org/). The pathway mapping for the k number was conducted using the Kyoto Encyclopedia of Genes and Genomes (KEGG) Automatic Annotation Server (KAAS, version 2.1) (13), and pathway enrichment (Hypergeometric test p < 0.05 and q < 0.05) was conducted against Arabidopsis and Populus using KOBAS (version 3.0) (14).

### **Isolation, maintenance, and characterization of Rhizobia from rhizosphere soil and root nodules of greenhouse-grown P. cineraria plants**

Surface sterilized seeds *of P. cineraria* were germinated in 8x8cm pots with dirt collected from the rhizosphere of the desert tree and a sterile potting mix at a 1:1 ratio. Pots were maintained in saturated water in the greenhouse at 24^o^C, 65% humidity with 18 hr. light, 8hr dark cycle. The seedlings were harvested after 6-8 weeks. The soil was removed by washing the roots, and the seedlings were photographed. Nodules from the seedling roots were randomly selected to isolate associated microbes.

Roots and nodule samples collected from young plants growing on the desert farm or growth chamber were first pooled and crushed in 10 mM Tris buffer pH 7.5, and cell debris was removed by spinning at 800 rpm for 5 min. The supernatant, after centrifugation, was streaked on yeast extract-mannitol (YM) agar media with Congo Red and NFM Agar (15). Isolated colonies appearing after 3-5 days of incubation at 28^o^C were sub-cultured on the same media. The pure cultures derived from single colonies were stored in LB medium with 25% glycerol at -80^o^C.

Genomic DNA was isolated from pure cultures by growing them in 5ml YM broth for 48hr at 28^o^C with shaking at 250 rpm. The cells were harvested and washed twice with TE buffer at pH 7.5 by centrifugation at 6000 rpm for 5 min. Genomic DNA was isolated as previously described (16). For the initial characterization of pure cultures, 16s rRNA genes were amplified using universal 27F (5’-AGAGTTTGATCCTGGCTCAG-3)’ and 1492R (5’-GGTTACCTTGTTACGACTT-3’) and the following PCR parameters;95^o^C for 3 min, [94^o^C for the 90s., 53^o^C for 35s., 50^o^C for 1.5 min] X 35 followed by 72^o^C extension for 5 min. The PCR reaction products were excised from an agarose gel and purified using the Qiagen Gel purification kit; Purified PCR fragments were sent to Microgen for Sanger-sequencing.

**Supplementary Results**

*nif*H microbiome diversity:  The PCoA graph separated nodule samples based on the source tree genotypes (DT vs. FT) and nodule compartments (NSM vs. NEM) for each genotype (Fig. S7A). A total of 5 phyla, 33 genera, and 72 NOTUs were identified from representative NOTU sequences. Of 72 NOTUs, 18 NOTUs represented 99% of the total *nif*H microbiome in the *Prosopis* nodules. Members of Betaproteobacteria were most abundant on the surface (NSM) of the nodule. In contrast, Alphaproteobacteria were more dominant in the nodules' endophytic compartment (NEM) in both genotypes (Fig. 7B). An unclassified phylum represented by OTU7 was abundant in the DT root nodules of NSM samples. The genera *Ensifer* (represented by NOTU02, NOTU03, NOTU04, NOTU22, NOTU54, NOTU58) and *Herbaspirillum* (NOTU1) were the most dominant in our dataset. Ensifer NOTUs were distributed such that when one NOTU was low in any sample, it was supplemented by other NOTUs belonging to the same *Ensifer* genus (Supplementary data). Generally, *Ensifer* was significantly more abundant in FT-NEM samples than in FT-NSM samples. Besides, *Herbaspirillum* was more abundant in NSM samples than NEM samples (Fig. S7C). The phylogeny of NOTUs was assessed by NCBI BLAST analysis of reference sequences and subsequent phylogenetic analysis of the top ten hits of each OTU sequence (Fig. S8).

**Many cultivable bacteria from the roots belong to Actinobacteria and firmicutes, whereas rhizobia dominated root nodules.**

The roots and nodule samples of *P. cineraria* were collected from environmental and laboratory samples and crushed in a sterile TE buffer. The extract was streaked on agar plates containing YM with congo-red and NFM Agar. Isolated colonies, which appeared after three to four days of incubation at 28^0^C, were subsequently subcultured three to four times to obtain pure cultures. Isolates were then identified by sequencing the 16s RNA gene using universal primers 27F and 1492R. We sequenced 16S genes of 23 bacterial isolates from nodules and ten bacterial isolates from the root extracts and soil. All the bacterial isolates from nodule extracts belong to Alphaproteobacteria (Fig. S14).

In contrast, the root isolates are homologous to firmicutes (3 *Bacillus* sp. and 1 *Paenibacillus* sp.), Actinomycetes (4 *Streptomyces* sp. and 1 *Isoptericola* sp.), and Alphaproteobacteria (3 *Ensifer* sp.). The root nodule isolates were clustered into three major groups: Ensifer/Sinorhizobium group, Rhizobium group, and Agrobacterium and Rhizobium group. Five of the 23 bacterial isolates identified as Rhizobium species did not cluster into any of the three groups (Fig. S14). We selected six bacterial isolates to represent each phylogenetic cluster for its effect on root nodulation and plant growth.

Some other actinobacterial members such as OTU26 (*Nocordioides* sp.), OTU41 (Unclassified Actinobacteria), OTU55 (Unclassified Actinobacteria), OTU90 (Unclassified Actinobacteria), and OTU346 (Unclassified Actinobacteria) are overrepresented in the REE fractions. Members of the genus *Actinophytocola* were previously reported from the roots of a nitrogen-fixing leguminous tree, *Colophospermum mopane* (17), Thai glutinous rice plants (18) and *Maytenus austroyunnanensis* (19), etc.

**Supplementary discussion**

Among the top 10 MG-RAST functions enriched in the metagenomes, two were related to bacteriophage proteins. Bacteriophages were isolated and characterized from several SNF bacteria such as *Sinorhizobium meliloti* (20, 21), *Rhizobium leguminosarum* (22), *Rhizobium etli* (23), *Mesorhizobium* sp.(24), *Bradyrhizobiu*m sp. (25), etc. In the completely sequenced genomes of *Rhizobium etli* strains CFN42 and CIAT652 (26, 27), some open reading frames (ORFs) with relatively high degrees of identity to bacteriophage genes exist, such as DNA integrases, DNA transferases, lysozymes, and the small and large subunits of DNA terminase. Generally, phages have been used for typing *Rhizobium* strains as indicators of diversity and symbiotic performance (28, 29). They have also been helpful as genetic tools to construct chromosome linkage maps and allocate nitrogen fixation and nodulation loci in plasmids (Martin and Long, 1984). In some symbiotic interactions, such as *Bradyrhizobium*: Soybean interactions, it was shown that phage-resistant *Bradyrhizobium* sp. performed better and improved the soybean seed yield (29). Studying further mechanisms by which phage resistance improves nodulation efficiency would be interesting.

The host plant genotype played a more significant role than the nodule compartments (NSM vs. NEM) in determining the relative abundance of the total microbiome and nifH microbiome in growth chamber-grown plants. This observation differs from root microbiome studies, where host genotype played a less significant role than the root compartments (e.g., Rh, REE, REN) (30, 31). Our observation is in line with at least one previous study in soybean, which showed that host genotypes played a significant role in the nodule microbiome composition (32).

The top NOTU in the *nif*H metagenome analysis is closely related to the genera *Herbaspirellum*, also found in the 16S, metagenomic, and metatranscriptome data in less than 1% of the total microbiome. *Herbaspirillum* species were previously isolated from the root nodules of *Robinia pseudoacacia*, *Phaseolus vulgaris*, *Acacia* sp., etc., although the role of *Herbaspirillum* in the root nodulation has not been demonstrated (33-35). However, members of this genus are known to have functional nitrogenase enzymes and assist in associative nitrogen fixation in several plant species (35, 36).

Carbon and nitrogen are the most important components of all living organisms, and their metabolism is intimately linked. Tissue nitrogen level increased significantly in the presence of rhizobia in *P. cineraria* plants, though we did not find any significant increase in tissue carbon levels. It is known that there is an increase in photosynthetic rates by up to 28% due to rhizobial symbiosis (37). However, root respiration during active nitrogen fixation directly associated with nitrogenase activity will require between 3 to 5g C g-1 N (38). Our analysis also showed drought stress significantly affected root nodulation and plant growth. There was a significant increase in the above-ground biomass with increased water availability. However, root growth and nodulation enhanced with the increase in water availability up to 30% field capacity. Further, an increase in irrigation did not significantly increase root growth and nodule number. This is in line with old studies, where it was shown that mild drought treatments had no significant effect on nodule number or dry weight. Significant (P < 0.05) differences were observed in nodule number and dry weight only when severe drought was imposed (39). In the previous studies, several mechanisms, including carbon shortage and nodule carbon metabolism, oxygen limitation, and feedback regulation by the accumulation of N fixation products, were found to affect symbiotic nitrogen fixation (40).

**References**

1. Millonig G. 1976. Laboratory manual of biological electron microscopy. Saviolo.

2. Morris JK. 1965. A formaldehyde glutaraldehyde fixative of high osmolality for use in electron microscopy. J cell Biol 27:1A-149A.

3. Sjöstrand FS. 1956. The ultrastructure of cells as revealed by the electron microscope. International Review of Cytology 5:455-533.

4. Reynolds ES. 1963. The use of lead citrate at high pH as an electron-opaque stain in electron microscopy. Journal of cell biology 17:208-212.

5. Chaluvadi S, Bennetzen JL. 2018. Species-associated differences in the below-ground microbiomes of wild and domesticated setaria. Frontiers in plant science 9:1183.

6. Livak KJ, Schmittgen TD. 2001. Analysis of relative gene expression data using real-time quantitative PCR and the 2(-Delta Delta C(T)) Method. Methods 25:402-8.

7. Chen Y, Dong J, Bennetzen JL, Zhong M, Yang J, Zhang J, Li S, Hao X, Zhang Z, Wang X. 2017. Integrating transcriptome and microRNA analysis identifies genes and microRNAs for AHO-induced systemic acquired resistance in N. tabacum. Scientific reports 7:1-13.

8. Wang X, Liu B-y, Zhao Q, Sun X, Li Y, Duan Z, Miao X, Luo S, Li J. 2019. Genomic Variance and Transcriptional Comparisons Reveal the Mechanisms of Leaf Color Affecting Palatability and Stressed Defense in Tea Plant. Genes 10:929.

9. Sudalaimuthuasari N, Ali R, Kottackal M, Rafi M, Al Nuaimi M, Kundu B, Al-Maskari RS, Wang X, Mishra AK, Balan J, Chaluvadi SR, Al Ansari F, Bennetzen JL, Purugganan MD, Hazzouri KM, Amiri KMA. 2022. The Genome of the Mimosoid Legume Prosopis cineraria, a Desert Tree. Int J Mol Sci 23.

10. Pertea M, Kim D, Pertea GM, Leek JT, Salzberg SL. 2016. Transcript-level expression analysis of RNA-seq experiments with HISAT, StringTie and Ballgown. Nature protocols 11:1650.

11. Love MI, Huber W, Anders S. 2014. Moderated estimation of fold change and dispersion for RNA-seq data with DESeq2. Genome biology 15:550.

12. Li W, Xiang F, Zhong M, Zhou L, Liu H, Li S, Wang X. 2017. Transcriptome and metabolite analysis identifies nitrogen utilization genes in tea plant (Camellia sinensis). Scientific reports 7:1-12.

13. Moriya Y, Itoh M, Okuda S, Yoshizawa AC, Kanehisa M. 2007. KAAS: an automatic genome annotation and pathway reconstruction server. Nucleic acids research 35:W182-W185.

14. Xie C, Mao X, Huang J, Ding Y, Wu J, Dong S, Kong L, Gao G, Li C-Y, Wei L. 2011. KOBAS 2.0: a web server for annotation and identification of enriched pathways and diseases. Nucleic acids research 39:W316-W322.

15. Dahal B, NandaKafle G, Perkins L, Brözel VS. 2017. Diversity of free-Living nitrogen fixing Streptomyces in soils of the badlands of South Dakota. Microbiological Research 195:31-39.

16. Wilson K. 2001. Preparation of Genomic DNA from Bacteria. Current Protocols in Molecular Biology 56:2.4.1-2.4.5.

17. Burbano CS, Grönemeyer JL, Hurek T, Reinhold-Hurek B. 2015. Microbial community structure and functional diversity of nitrogen-fixing bacteria associated with Colophospermum mopane. FEMS microbiology ecology 91.

18. Indananda C, Matsumoto A, Inahashi Y, Takahashi Y, Duangmal K, Thamchaipenet A. 2010. Actinophytocola oryzae gen. nov., sp. nov., isolated from the roots of Thai glutinous rice plants, a new member of the family Pseudonocardiaceae. International journal of systematic and evolutionary microbiology 60:1141-1146.

19. Qin S, Chen HH, Zhao GZ, Li J, Zhu WY, Xu LH, Jiang JH, Li WJ. 2012. Abundant and diverse endophytic actinobacteria associated with medicinal plant Maytenus austroyunnanensis in Xishuangbanna tropical rainforest revealed by culture‐dependent and culture‐independent methods. Environmental microbiology reports 4:522-531.

20. Kowalski M, Małek W, Czopska-Dolecka J, Szlachetka M. 2004. The effect of rhizobiophages on Sinorhizobium meliloti - Medicago sativa symbiosis. Biology and Fertility of Soils 39:292-294.

21. Werquin M, Ackermann H-W, Levesque RC. 1988. A Study of 33 Bacteriophages of Rhizobium meliloti. Applied and environmental microbiology 54:188-196.

22. Kankila J, Lindström K. 1994. Host range, morphology and dna restriction patterns of bacteriophage isolates infecting Rhizobium leguminosarum bv. trifolii. Soil Biology and Biochemistry 26:429-437.

23. Santamaría RI, Bustos P, Sepúlveda-Robles O, Lozano L, Rodríguez C, Fernández JL, Juárez S, Kameyama L, Guarneros G, Dávila G, González V. 2014. Narrow-Host-Range Bacteriophages That Infect <span class="named-content genus-species" id="named-content-1">Rhizobium etli</span> Associate with Distinct Genomic Types. Applied and Environmental Microbiology 80:446-454.

24. Turska-Szewczuk A, Russa R. 2000. A new Mesorhizobium loti HAMBI 1129 phage isolated from Polish soil. Curr Microbiol 40:341-3.

25. Appunu C, Dhar B. 2008. Isolation and symbiotic characteristics of two Tn5-derived phage-resistant Bradyrhizobium japonicum strains that nodulate soybean. Curr Microbiol 57:212-7.

26. González V, Acosta JL, Santamaría RI, Bustos P, Fernández JL, Hernández González IL, Díaz R, Flores M, Palacios R, Mora J, Dávila G. 2010. Conserved Symbiotic Plasmid DNA Sequences in the Multireplicon Pangenomic Structure of <em>Rhizobium etli</em>. Applied and Environmental Microbiology 76:1604-1614.

27. Santamaría RI, Bustos P, Sepúlveda-Robles O, Lozano L, Rodríguez C, Fernández JL, Juárez S, Kameyama L, Guarneros G, Dávila G, González V. 2014. Narrow-host-range bacteriophages that infect Rhizobium etli associate with distinct genomic types. Applied and environmental microbiology 80:446-454.

28. Bromfield ESP, Tambong JT, Cloutier S, Prévost D, Laguerre G, van Berkum P, Thi TVT, Assabgui R, Barran LR. 2010. Ensifer, Phyllobacterium and Rhizobium species occupy nodules of Medicago sativa (alfalfa) and Melilotus alba (sweet clover) grown at a Canadian site without a history of cultivation. Microbiology (Reading, England) 156:505-520.

29. Jaiswal SK, Anand A, Dhar B, Vaishampayan A. 2012. Genotypic Characterization of Phage-Typed Indigenous Soybean Bradyrhizobia and Their Host Range Symbiotic Effectiveness. Microbial Ecology 63:116-126.

30. Chaluvadi S, Bennetzen JL. 2018. Species-Associated Differences in the Below-Ground Microbiomes of Wild and Domesticated Setaria. Front Plant Sci 9:1183.

31. Edwards J, Johnson C, Santos-Medellín C, Lurie E, Podishetty NK, Bhatnagar S, Eisen JA, Sundaresan V. 2015. Structure, variation, and assembly of the root-associated microbiomes of rice. Proceedings of the National Academy of Sciences 112:E911-E920.

32. Sharaf H, Rodrigues RR, Moon J, Zhang B, Mills K, Williams MA. 2019. Unprecedented bacterial community richness in soybean nodules vary with cultivar and water status. Microbiome 7:63.

33. Fan M-C, Guo Y-Q, Zhang L-P, Zhu Y-M, Chen W-M, Lin Y-B, Wei G-H. 2018. Herbaspirillum robiniae sp. nov., isolated from root nodules of Robinia pseudoacacia in a lead–zinc mine. International journal of systematic and evolutionary microbiology 68:1300-1306.

34. Hoque MS, Broadhurst LM, Thrall PH. 2011. Genetic characterization of root-nodule bacteria associated with Acacia salicina and A. stenophylla (Mimosaceae) across south-eastern Australia. International Journal of Systematic and Evolutionary Microbiology 61:299-309.

35. Valverde A, Velázquez E, Gutiérrez C, Cervantes E, Ventosa A, Igual J-M. 2003. Herbaspirillum lusitanum sp. nov., a novel nitrogen-fixing bacterium associated with root nodules of Phaseolus vulgaris. International Journal of Systematic and Evolutionary Microbiology 53:1979-1983.

36. Pedrosa F, Benelli E, Yates M, Wassem R, Monteiro R, Klassen G, Steffens M, Souza E, Chubatsu L, Rigo L. 2001. Recent developments in the structural organization and regulation of nitrogen fixation genes in Herbaspirillum seropedicae. Journal of biotechnology 91:189-195.

37. Kaschuk G, Kuyper TW, Leffelaar PA, Hungria M, Giller KE. 2009. Are the rates of photosynthesis stimulated by the carbon sink strength of rhizobial and arbuscular mycorrhizal symbioses? Soil Biology and Biochemistry 41:1233-1244.

38. Minchin FR, Witty JF. 2005. Respiratory/carbon costs of symbiotic nitrogen fixation in legumes, p 195-205, Plant respiration. Springer.

39. Sinclair TR, Zimet AR, Muchow RC. 1988. Changes in soybean nodule number and dry weight in response to drought. Field Crops Research 18:197-202.

40. Serraj R. 2003. Effects of drought stress on legume symbiotic nitrogen fixation: physiological mechanisms. Indian J Exp Biol 41:1136-41.
