# Supplementary material for: Microbiome properties in the root nodules of Prosopis cineraria, a leguminous desert tree
Source: Microbiol Spectr. 2024 Apr 16;12(6):e03617-23. doi: 10.1128/spectrum.03617-23 (PMC11237379; doi:10.1128/spectrum.03617-23)
Supplement: Table S6 — qPCR primer list. [file spectrum.03617-23-s0010.docx]

Supplementary table 6: qPCR primer list

| Organism | Target gene | Primes Sequence | | Amplicon Size in bp |
| --- | --- | --- | --- | --- |
| *Prosopis cineraria* |  | Forward | Reverse |  |
|  | NF-Y | TCGGGTTCAATCAGCCTATG | AATCGGTCCATCGTCAGTTG | 137 |
|  | GAPD | GAGGAGCAAGGCAATTGGTA | GTTGTGATTAGGGTGGTGCT | 150 |
|  | STP | TCTTGGGTCGTATATTGCTTGG | GTGGCGTAGTTAATGAAGTTGG | 166 |
|  | PIP21 | ACCGGTTTAGGCGCTGAGAT | AAGACTCCTGGCCGGGTTG | 195 |
| *Sinohizobium* sp. | NifH | TTCCCTGCGTTCGCTTCTTATC | TGCGAATGAGCTTTGATCCATGGAT | 125 |
| *Ensifer* sp. | NifH | TCCACTTCATTCCTCATTGCCACT | GAAGCGAACGCAGGGAACTG | 135 |
| *Mesorhizobium* sp. | NifH | ATGGAATGTTGCCCCGGTTCAAG | CTGCTCGACTTCGGCATCATGA | 132 |

glyceraldehyde-3-phosphate dehydrogenase (*GAPD*), Sugar transporter protein (STP), plasma membrane intrinsic protein (PIP2 isoform), betaine aldehyde dehydrogenase (BADH), nitrogenase coding gene (nifH ), and nuclear factor Y transcription factors (NF-YA).
